# High diversity in the regulatory region of Shiga toxin encoding bacteriophages

**DOI:** 10.1186/s12864-022-08428-5

**Published:** 2022-03-24

**Authors:** Annette Fagerlund, Marina Aspholm, Grzegorz Węgrzyn, Toril Lindbäck

**Affiliations:** 1 Norwegian Institute of Food, Fisheries and Aquaculture Research, Ås, Norway; 2 Department of Paraclinical Sciences, Faculty of Veterinary Medicine, Norwegian University of Life Sciences, Ås, Norway; 3 Department of Molecular Biology, Faculty of Biology, University of Gdañsk, Gdañsk, Poland

**Keywords:** EHEC, Stx phage, Bacteriophage genetics, Lysogen, Lytic, Phage replication

## Abstract

**Background:**

Enterohemorrhagic *Escherichia coli* (EHEC) is an emerging health challenge worldwide and outbreaks caused by this pathogen poses a serious public health concern. Shiga toxin (Stx) is the major virulence factor of EHEC, and the *stx* genes are carried by temperate bacteriophages (Stx phages). The switch between lysogenic and lytic life cycle of the phage, which is crucial for Stx production and for severity of the disease, is regulated by the CI repressor which maintain latency by preventing transcription of the replication proteins. Three EHEC phage replication units (Eru1-3) in addition to the classical lambdoid replication region have been described previously, and Stx phages carrying the Eru1 replication region were associated with highly virulent EHEC strains.

**Results:**

In this study, we have classified the Eru replication region of 419 Stx phages. In addition to the lambdoid replication region and three already described Erus, ten novel Erus (Eru4 to Eru13) were detected. The lambdoid type, Eru1, Eru4 and Eru7 are widely distributed in Western Europe. Notably, EHEC strains involved in severe outbreaks in England and Norway carry Stx phages with Eru1, Eru2, Eru5 and Eru7 replication regions. Phylogenetic analysis of CI repressors from Stx phages revealed eight major clades that largely separate according to Eru type.

**Conclusion:**

The classification of replication regions and CI proteins of Stx phages provides an important platform for further studies aimed to assess how characteristics of the replication region influence the regulation of phage life cycle and, consequently, the virulence potential of the host EHEC strain.

**Supplementary Information:**

The online version contains supplementary material available at 10.1186/s12864-022-08428-5.

## Background

Enterohemorrhagic *Escherichia coli* (EHEC) is an important foodborne pathogen, responsible for disease in humans ranging from uncomplicated diarrhea to severe conditions such as hemorrhagic colitis and hemolytic uremic syndrome (HUS) [1–3]. WHO has estimated that 10% of patients with EHEC infection develop HUS and the most important sources for HUS cases were contaminated beef [4]. The first major EHEC outbreak found place in U.S.A. in 1982 and was caused by hamburgers contaminated by *E. coli* O157:H7 strain EDL 933 carrying the Stx2 phage 933W. Since then, the world has experienced multiple outbreaks of EHEC disease involving other serotypes than O157:H7 and new variants are constantly emerging [5, 6]. Shiga toxin (Stx) is the major virulence factor of EHEC, and it exists in two distinct forms, Stx1 and Stx2. Each form comprises several subtypes [7] where some subtypes such as Stx2a are associated with severe disease while Stx2c is considered less potent [8, 9].

The genes encoding Stx are carried by temperate bacteriophages (Stx phages) [10]. After infection, Stx phages follow either a lysogenic or lytic pathway. The lysogenic pathway involves integration of phage DNA into the host genome and replication of the phage genetic material along with the chromosome of the host cell. The lytic pathway leads to proliferation of the Stx phage, death of the host bacterial cell and release of new phage particles [11]. Induction of the lytic pathway is also accompanied by production and release of substantial amounts of Stx toxin. Experimental infections of microbiome-repleted mice suggest that Stx prophage induction, but not production of phage particles, is required for development of lethal disease [12]. As the amount of produced Stx influences the severity of the disease, the mechanisms regulating the switch from lysogenic to lytic life cycle is highly relevant for the pathogenicity of the host *E. coli* strain.

Since the first sequenced Stx phages shared substantial genomic similarity to phage lambda it has been assumed that they behave similarly [13, 14]. The increasing availability of whole genome sequences has revealed that Stx-encoding prophages are very diverse and, sometimes, exhibit only limited similarity towards phage lambda [15]. We have previously reported Stx phages with non-lambdoid replication regions and named the regions Eru (EHEC phage replication unit) [15]. The non-lambdoid Stx phages completely lack the *O* and *P* genes, encoding proteins involved in replication initiation of the lambdoid phage genome, and instead carry genes which have previously not been described in connection to replication of Stx phages. Three non-lambdoid Stx phage replications, Eru1-3, have so far been described [15]. One of the Eru types, Eru1, is carried by the highly pathogenic EHEC strains that caused the Norwegian O103:H25 outbreak in 2006 and the large O104:H4 outbreak in Europe in 2011. It was also shown that Eru1 phages exhibited a less stable lysogenic state than the classical lambdoid Stx phages, which could increase the pathogenicity of the host *E. coli* strain [15]. The majority of EHEC strains carrying Eru1, Eru2 and Eru3 type of Stx phages were US isolates whose genome sequences were submitted to NCBI databases by the United State Department of Agriculture, the US Food and Drug Administration, and the Food-borne Pathogen Omics Research Center.

**Fig. 1:**
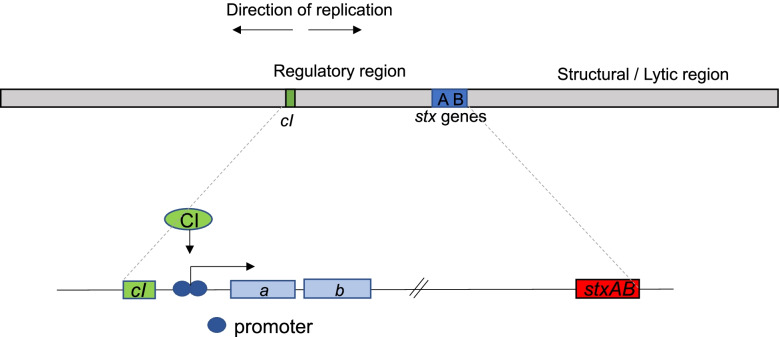
A schematic overview of the genome of an Stx phage. The boxes labeled *a* and *b* indicate the replication genes which are represented by *O* and *P* in phage lambda and by other less characterized genes in Eru1-3 [15]

Despite the high genetic diversity among Stx phage genomes, the phage replication region and the lysis-lysogeny regulatory systems are always located upstream and in the vicinity to the *stxA* gene [16]. This region mediates the switch between repression and induction of the prophage, and the mechanisms regulating these events have been studied in detail in phage lambda. The key elements responsible for regulating the life cycle of phage lambda are the gene encoding repressor CI (*cI*), the promoter binding the CI repressor and the adjacent upstream genes, transcribed in the opposite direction of *cI* (Fig. 1) [17, 18]. The lambda CI repressor downregulates expression of genes involved in production of new phage particles, i.e., the lytic cycle, by specific binding to the promoter region of the adjacent genes encoding the O and P proteins which initiate replication of the lambda genome [19]. The crystal structure of CI has been solved and revealed that the protein is functional as a homodimer and that repression occurs when two subunits bind cooperatively to adjacent operator sites on the DNA [20]. The C-terminal domain mediates the dimer formation and the dimer-dimer interactions enable CI to bind cooperatively to two or more operator sites [21, 22] while the N-terminal domain contains a helix-turn-helix DNA-binding domain [23, 24]. Upon DNA damage, the SOS-response protein RecA becomes activated and may in lysogenic cells stimulate autocleavage of CI [25]. Cleaved CI can no longer bind to DNA and its repression of the promoters in the replication module is thus relieved. In lambdoid prophages, repression by CI ultimately controls Shiga toxin production and release of CI is required for Stx production [26, 27]. A lysogenic derivate of the Stx phage 933W, encoding a non-cleavable CI repressor, was found unable to produce Stx [26]. It has also been demonstrated that low lysogenic stability coincidences with low intracellular levels of the CI repressor [28].

Some EHEC strains appear more virulent than others and the type of Stx produced is known to contribute significantly to the pathogenicity of the EHEC strain [9] but the amount of toxin produced should also be considered. The increasing number of outbreaks of gastrointestinal disease and HUS caused by EHEC have stimulated studies on the Stx phages to better understand their contribution to the pathogenicity of the host *E. coli* strain. However, there is still very limited knowledge on how the different types of replication regions seen among the Stx phages influences the stability of the lysogenic state and the switch to lytic cycle. In this study, we have classified the CI repressor sequences of 260 Stx phages into clade I-VIII and their replication regions into 13 Eru types to provide a platform for further studies of how the genetic structure of the Stx prophages influences the virulence potential of the host EHEC strain.

## Results

**Fig. 2:**
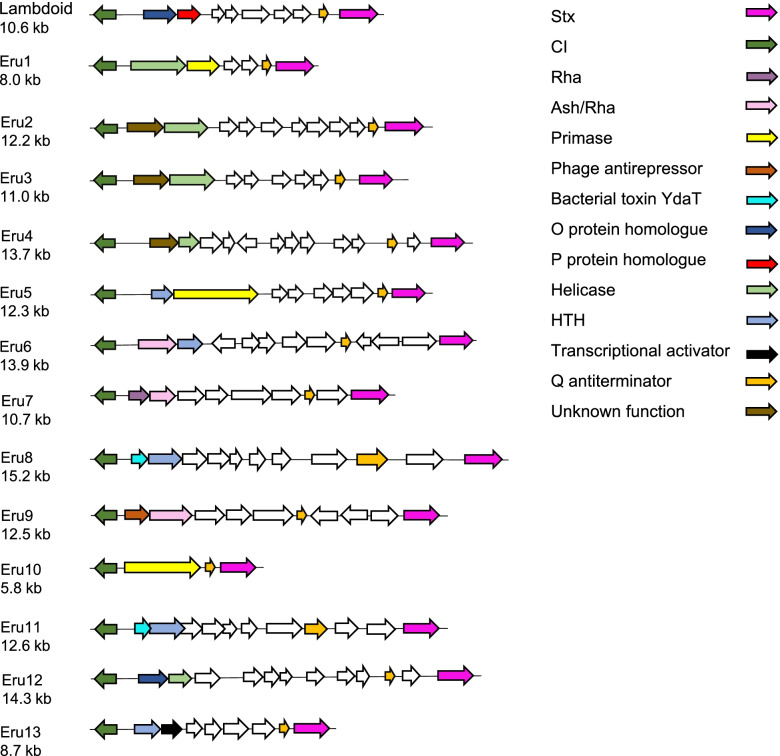
Physical maps of the region between *cI* (green) and *stx* (pink). The color code also indicates the putative function of the proteins encoded by the genes directly upstream of *cI*. White arrows indicate open reading frames (ORFs) which are not discussed in this study

Eru types were defined by the identity of the proteins encoded by the two genes located directly upstream and in opposite direction of *cI* regardless of their function. The identity of the remaining proteins between *cI* and *stx* were not considered in this study. Four novel Eru phage types (Eru4-7) were detected among 120 Stx-converting phage genomes retrieved from NCBI virus database (Fig. 2; Additional file 1), while an additional six novel Eru types, Eru8 to Eru13, were detected among 298 genome sequences obtained from ten examined NCBI BioProjects (Fig. 2; Additional file 2). These genome sequences comprise both EHEC isolated from patients and Shiga toxin producing *E. coli* (STEC) isolated from other sources. The genomes are available as unfinished genome assemblies and only sequences where both *cI* and *stx* were located on same contig were included in this study. Eru2 and Eru3, described in a previous study, both carry genes encoding a protein of unknown function and a helicase directly upstream of *cI* [15]. However, since the two unknown proteins share a low sequence identity (10%), phages carrying these protein combinations were still assigned to different Eru types [15]. Phages representing each Eru type are listed in Table 1 as reference phages for each Eru type.

**Table 1:** Accession numbers of sequences representing each Eru type

Name	Representative phage^b^	NCBI accession no	Stx type	Origin	Year
Lambdoid^a^	933W	NC_000924	Stx2	USA	1982
Eru1^a^	TL-2011c	NC_019442	Stx2	Norway	2006
Eru2^a^	TW14359	NC_013008	Stx2	USA	2006
Eru3^a^	VT2-Sakai	AP000422	Stx2	Japan	1996
Eru4^b^	Shigella phage 75/02	NC_029120.1	Stx1	Hungary	2013
Eru5	Stx2-converting phage Stx2a_F403	AP012529.1	Stx2	Japan	2012
Eru6	Stx2-converting phage Stx2a_WGPS2	AP012537.1	Stx2	Japan	2012
Eru7	Stx2a-converting phage Stx2_14040	LC567818.1	Stx2	Japan	2020
Eru8		LODB01000401.1	Stx2	Netherlands	2013
Eru9		JAGEXB010000044	Stx2	Germany	2018
Eru10		LOGT01000177.1	Stx2	Netherlands	2013
Eru11		LOIJ01000033.1	Stx2	Netherlands	2013
Eru12		CCVP01000073.1	Stx1	Norway	2009
Eru13		AATHWC010000004.1	Stx1	England	2020

^a^Eru types previously described in Llarena et al., 2021 [15]

^b^The Eru4 type is represented by an *stx1* carrying phage from a clinical isolate of *Shigella sonnei* from Hungary while the rest are prophages found in *E. coli *[29]

The distribution of Eru types found among the 120 sequenced Stx phages are shown in Table 2.

**Table 2:** Number of Eru types in the data set of 120 Stx-converting phage genomes retrieved from the NCBI virus database (taxid:10,239)

Eru type	Number	Reference
Lambdoid	9	[15]
Eru1	7	[15]
Eru2	57	[15]
Eru3	18	[15]
Eru4	2	This Study
Eru5	4	This study
Eru6	3	This study
Eru7	20	This study

The relatively high number of phage genomes belonging to Eru2, Eru3 and Eru7 could be due to a bias related to the number of deposited sequences from different studies (Additional file 1).

### Distribution of Eru types among Stx phages from Western Europe

The national distribution of Eru types found among 298 identified contigs carrying both *stx* and *cI* from ten European BioProjects is shown in Table 3.

**Table 3:** Distribution of the thirteen Eru types (1–13) and the lambdoid (L) type in ten European BioProjects

Bioproject ID (NCBI)	Country	Source	No of samples^a^	Stx type	Eru type													
					**L**	**1**	**2**	**3**	**4**	**5**	**6**	**7**	**8**	**9**	**10**	**11**	**12**	**13**
PRJNA285020	Netherlands	Human	134	Stx1		5	3			30	1		2					
				Stx2	4						10	13	1		2	2		
PRJEB6447	Norway	Human	97															
				Stx1	1	4			10	1	2						1	
				Stx2	1	12	1				6	14			2		2	1
PRJNA680568	Switzerland	Human	19															
				Stx1														
				Stx2								12						
PRJNA694525	Switzerland	Dog/cat fodder	32															
				Stx1		2			8									
				Stx2	1	1					1		2		2			
PRJNA438214	Switzerland	Human	18															
				Stx1														
				Stx2								15						
PRJNA248042	England	Human	> 3000															
				Stx1	37	3			3							1		1
				Stx2		23	5			2		26						
PRJNA70699	France	Human	8															
				Stx1	1													
				Stx2							1	2						
PRJNA715185	Germany	Flour	56															
				Stx1														
				Stx2										5				
PRJNA666781	Italy	Raw milk cheese	7															
				Stx1								4						
				Stx2														
PRJNA643688	Portugal	Cattle	12															
				Stx1	1						3	1						
				Stx2		1						1	2					
Total number sample of each Eru type^b^				Stx1	**40**	**14**	3	0	**21**	**31**	**6**	**5**	2	5	0	1	1	1
				Stx2	**6**	**37**	6	0	**0**	**2**	**18**	**83**	5	0	6	2	2	1

^a^ “No of samples” refer to the number of samples included in the different Bioprojects, however, only contigs containing both *cI* and *stx* were Eru typed

^b^The numbers of the more common types are indicated in bold in the bottom rows

The distribution of Eru types indicates that the lambdoid and the Eru1, 4, 5, 6 and 7 phage types are among the most common types of Stx phages in Europe, and that Eru7 appears to be particularly widespread (Table 3). The lambdoid- and the Eru4 and Eru5 phage types seem more inclined to carry genes encoding Stx1, while Eru1, Eru6 and Eru7 seems more often associated with genes encoding Stx2 (Table 3).

### The Eru proteins

All Eru phages carry genes encoding different types of DNA binding proteins, such as helicases, primases, or other helix-turn-helix (HTH) motif proteins, in the first and/or second position directly upstream of *cI* (Fig. 2). Eru6, Eru7 and Eru9 phages carry genes encoding proteins of the Phage_pRha protein family (pfam09669) directly upstream of *cI* (Fig. 2). The Rha domain, which contain a winged helix-turn-helix DNA-binding motif, is also found in other temperate phages where it has been suggested to have phage regulatory function [30, 31]. Some of the Rha proteins also contain the Ash domain (PF10554), which is present in the ASH protein of bacteriophage P4. However, no function has so far been assigned to this domain [31]. Eru4, and the previously described Eru2 and Eru3 [15], encode proteins of unknown function directly upstream of *cI* (Fig. 2). However, there are no similarities between these proteins, and they do not share any previously described protein domains. The primases encoded by genes carried by Eru1, Eru5 and Eru10 phages do not share any sequence similarities (< 10% amino acid identity). The amino acid sequence of the putative helicases encoded by Eru4 and Eru12 are 97% identical and they both share the AAA motif (PF13604) with the Eru1 helicase [15]. However, the overall sequence homology between the Eru4 and Eru12 helicases and the Eru1 helicase are low (< 10% amino acid identity).

Genes encoding HTH domain proteins are found in either the first or second position directly upstream of *cI* in Eru5, Eru6, Eru8, Eru11 and Eru13 (Fig. 2). The HTH proteins of Eru5 and Eru6 are 50% identical with a coverage of 66%, the HTH proteins of Eru8 and Eru13 are 59% identical over the total protein sequence, and all five proteins exhibit the HTH_36 motif (PF13730). The HTH proteins of Eru6 and Eru13 also share a motif (PF13814) which is found in protein families essential for relaxation and replication of plasmid DNA [32, 33]. Both Eru8 and Eru11 phages carry a gene encoding a protein with homology to the bacterial toxin YdaT (PF06254) directly upstream of *cI*. However, the two Eru-encoded toxin-like proteins share only 34% identity with each other. The shortest distance between *cI* and *stx* was displayed by Eru10, which only carried a bifunctional DNA primase-polymerase motif protein (PF09250) [34] and the Q antiterminator protein [35, 36] in this region. All other Eru phages also carried the gene encoding the antiterminator Q protein between *cI* and *stx*, indicating that this protein is essential for Stx phages.

### Eru types in particularly virulent EHEC

To explore the distribution of Eru types carried by highly pathogenic EHECs within a country we examined the Stx phages from six highly pathogenic EHEC O157:H7 strains that have caused larger outbreaks in the UK [36]. Four different Eru phage types in addition to the lambdoid type were found among the six strains (Table 4).

**Table 4:** Eru type of Stx phages of highly pathogenic EHEC O157:H7 isolates from UK

Strain ID	Reference	NCBI accession no	Year	Eru type
E30228	[37]	VXJO00000000	1983	Stx2a Eru5 Stx1a lambdoid
E34500	[38]	VXJN00000000	1983	Stx2a Eru1 Stx2c Eru2
E45000	[39]	VXJM00000000	1987	Stx2a Eru2
E116508	[39]	VXJP00000000	1996	Stx2a Eru5 Stx2c Eru2
315,176	[40]	VXJQ00000000	2014	Stx2a Eru2
267,849	[41]	VXJR0000000	2016	Stx2a Eru7 Stx2c Eru2

Among this panel of phages, all carrying *stx2c* and one carrying *stx2a* are of Eru2 type. Two *stx2a* carrying phages are of the Eru5 type, while the two remaining *stx2a* phages are of types Eru1 and Eru7. The only *stx1a* carrying phage among these isolates has a lambdoid replication region. Among the 97 Norwegian STEC strains in BioProject PRJEB6447, 15 strains caused HUS [42] and 13 of these strains carried *stx2* phages of Eru types 1, 2 or 7 (Additional file 2).

### The CI repressors

**Fig. 3:**
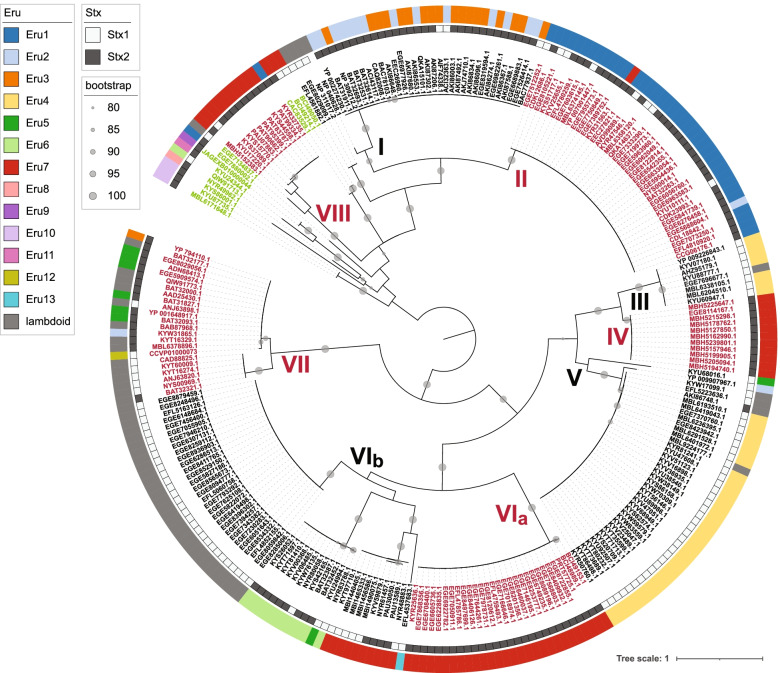
Maximum-likelihood phylogeny of 260 CI protein sequences. The tree was midpoint rooted and bootstrap values > 80% are indicated by grey circles. The Stx type is shown in the inner ring and the Eru type is shown in the outer ring. Clades that are discussed in the text are labelled with roman numerals

**Fig. 4:**
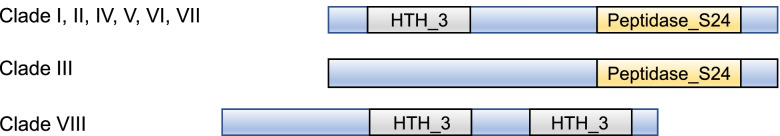
Domain structures of Stx phage CI repressors of Clade I-VIII. HTH_3 domains (grey) and Peptidase_S24 domains (yellow) were assigned according to Pfam

The CI repressor regulates transcription of the genes encoding the replication proteins defining the Eru type, so to further examine the replication region of Stx phages, a total of 260 annotated CI sequences (Additional file 3) were extracted from the phage genomes and used to build a phylogeny (Fig. 3). This analysis grouped the CI proteins into several distinct clades, for which major clades defined by less than 52% sequence identity were named I to VIII. Despite the higher sequence identity between Clade III and IV they are divided into two different clades as there were profound structural differences between the two clades for instance that Clade III completely lack the HTH binding domain. The CI protein from lambda phage (NP_040628.1) was most closely related to the CI proteins from phages of Eru types 2 and 3, all belonging to Clade I. The CI proteins of Eru2 and Eru3 phages in this clade were all identical and show an overall identity of 61% towards lambda CI. Lambda CI contains two protein domains, a HTH_3 domain [43] and a peptidase_S24 domain, which executes the CI autolysis [44, 45]. The two domains are conserved within the CI proteins belonging to Clades I, II, IV, V, VI and VII (Fig. 4). However, the CI proteins of Clade III and YP_009907967.1 in Clade V lack the HTH domain, while Clade VIII CI proteins lack the peptidase domain and instead exhibit an additional HTH domain (Fig. 4).

**Fig. 5:**
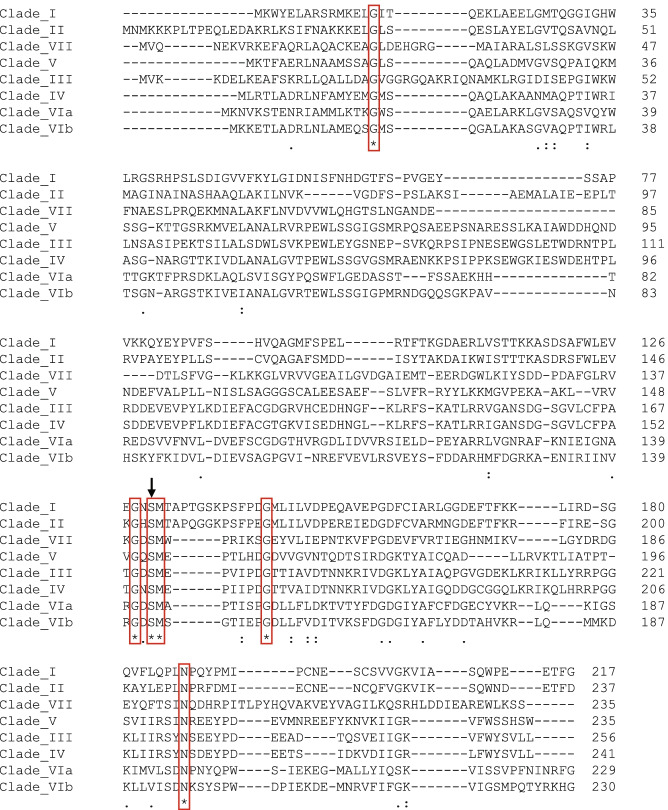
Sequence alignment of Clade I-VII Stx phage CI sequences. CI protein from Clade VIII is not included in the alignment due to large structural differences (see Fig. 4). Red boxes indicate the six amino acids that were conserved throughout all clades and the black arrow indicates the CI autocleavage residue found in this type of repressors [21]

In contrast to the observed high homology between CI proteins within a clade, the homology between the clades was low (Additional file 4). The highest CI homology was seen between Clades I and II (51%) and between Clades III and IV (60%). An amino acid sequence alignment of CI sequences from Clades I to VII is shown in Fig. 5. The alignment revealed six amino acids conserved throughout all clades, one of which was the lambda CI autocleavage residue S150 [21].

### Strong correlation between CI Clades and Eru type

There is a strong coherence between CI clades and Eru types which is not unexpected in light of their neighboring location in the phage genome. CI proteins belonging to Clades III and V are almost exclusively co-present with Eru4 replication proteins and the lambdoid replication type is mostly found in connection with Clades VIb and VII (Fig. 3). Similarly, the genes encoding CI proteins belonging to Clade II are almost exclusively located directly upstream of the genes defining Eru1, while those belonging to Clade I are located upstream of Eru2 and Eru3 (Fig. 3). However, a specific CI clade are not necessarily restricted to a specific Eru type and may regulate expression of different Eru types (Fig. 3). CI proteins of Clades III, V and VIb are linked to the lambdoid or Eru4 types and are mainly found in Stx1 producing phages (Fig. 3).

## Discussion

The present study shows that the replication region of Stx phages are genetically much more diverse than previously anticipated. This finding is important as differences in phages replication modules may influence the stability of the lysogenic state and the pathogenic potential of the host *E. coli* strain [15]. The Eru type was in the present study based on the type of proteins encoded by the two genes located directly upstream of *cI*. This definition is less differentiating than the definition used by Llarena et al. [15] where the entire region between *cI* and *stx* was considered. Due to the large variation of genes located between *cI* and *stx*, revealed in this study, we found that defining Eru type based on the identity of the two genes upstream of *cI* set the discrimination level to an appropriate level of sensitivity. However, it is very likely that additional proteins located in the region between *cI* and *stx* are required for replication of the phage.

Stx phages have traditionally been classified into the group of lambdoid phages based on similarity in behavior, genetic structure, and regulatory system. In phage lambda and lambdoid Stx phages, the assembly of the replication complex has been studied in detail [46] but there is so far no knowledge about the proteins involved in the replication process of Eru phages. Eru7 seems to be the most widespread Eru type in Europe and, together with Eru6 and Eru9, they encode proteins containing Rha or Rha/Ash domains. Rha domain proteins are common among temperate bacteriophages and large eukaryotic DNA viruses and is suggested to function as a regulatory protein that is involved in controlling the switch between lytic and lysogenic lifestyle [47]. Ash domain proteins are also common among bacteriophages, but little is known about their function [30, 31, 48]. However, none of these proteins have previously been associated with replication of Stx phages and it is of great interest to examine this aspect especially since Eru7 Stx phages seems to be among most common Eru types.

In phage lambda and lambdoid Stx phages, the CI repressor regulates expression of the *O* and *P* replication genes [18]. The *cI* gene is also present in the genomes of Eru phages suggesting that a similar regulatory mechanism is at play in non-lambdoid Stx phages. The genes located directly upstream of *cI* varies extensively between different Eru types, although most of them encode DNA binding proteins such as helicases, primases or other HTH motif proteins. When exploring the different Eru types, we observed that the amino acid sequence of the CI repressor differed substantially between Stx phages but there were also homologies which were used to group them into eight major Clades (I-VIII). In phage lambda, CI represses expression of upstream genes by forming dimers which bind to specific promoter sequences and self-cleavage of CI relieves the repression [20, 25]. All CI proteins belonging to Clade I-VII exhibit the self-catalytic Peptidase_S24 domain and the lambda S150 autocleavage residue [21, 44, 49] which mediates the cleavage of CI resulting in relieve of repression of the promoters in the replication module.

However, CI sequences belonging to Clade VIII lack this domain and it remains unexplored how this atypical CI protein is involved in regulating phage replication. Another atypical CI protein, lacking the HTH DNA-binding domain, was observed in Clade III, and the regulatory functions of this protein is also unknown. Considering the likelihood that CI is involved in regulation of upstream genes, the differences in amino acid sequence observed between CI repressors of different Eru types may reflect adaptation of binding specificities to match distinct target sequences. It is also likely that the differences observed between CI repressors may influence their regulatory network which, in turn, may influence the stability of the lysogenic state and the pathogenic potential of the host EHEC strain.

Stx phages are known to be highly mosaic and composed of gene segments with different evolutionary histories acquired through a variety of mechanisms, such as homologous recombination, transposition, and site-specific recombination [50–52]. The variation in CI protein sequence and Eru types and the different combinations of these revealed in the present study, indicates that Stx phages continuously change and that their classification may be less restricted to specific serotypes than previously anticipated [15]. We have previously suggested that the Eru2 type may be restricted to serotype O157:H7 and is predominant for the less potent subtype Stx2c phages [15]. However, we observed that among the 63 Eru2 phages detected in this study, fourteen were carried by *E. coli* of serotype O157:H7, while the remaining 49 phages (48 in Japanese EHEC strains (Additional file 1) and one in a Dutch EHEC strain (PRNJA285020 strains STEC 564; Additional file 2)) were carried by *E. coli* of serotype O121:H19. All Eru2 phages carried by O121:H19 strains encoded Stx2a, while all the O157:H7 strains carried Eru2 phages encoding Stx2c. We also observed that five of the six highly pathogenic strains of serotype O157:H7, which have caused large outbreaks in the UK carry Eru2 phages, and that four of these Eru2 phages encode Stx2c (Table 4). Although, the UK outbreak strains also do carry phages encoding the more potent Stx2a in addition. All in all, this indicate that Eru2 phages are not restricted to hosts of serotype O157:H7 but Eru2 phages carried by this serotype predominantly encode the Stx2c subtype.

Surprisingly, we did not observe any Eru3 type Stx phages among the European STEC strains examined during this study (Table 3). We have previously shown that Eru3 phages were carried by both serotype O157:H7 and O111 strains and often encode the potent subtype Stx2a [15]. A majority of the Eru3 type of Stx phages described in the previous work were isolated in the US, indicating that this phage type may be more widespread on the American continent than in Europe.

*E. coli* may carry multiple *stx* negative prophages with similarities to Stx phages together with multiple Stx phages in its genome [53]. Therefore, identification of the Eru type requires that the *stx* genes and the phage replication region is present on the same contig or scaffold. Assessment of Eru type from genome sequences generated by short read sequencing technology is often impossible due to contig breaks in the region between *cI* and *stx* (ND in Additional file 2). Stx phages often carry repetitive tRNA encoding genes immediately upstream of the *stx* making assembly of contigs difficult in this region.

In the present study, we observe that the Stx2a encoding phages carried by highly virulent EHEC strains from UK [39] and the HUS causing strains from Norway [42] are of Eru1-, Eru2-, Eru5- and Eru7-types. We have previously shown that the Eru1 type is carried by highly pathogenic EHEC strains and that Eru1 phages exhibit a less stable lysogenic state than the classical lambdoid Stx phages [15]. It is already well known that the outcome of EHEC disease is often more severe when the infection is caused by an *E. coli* strain producing Stx2 compared to a strain producing Stx1 [7, 9]. We must, however, emphasize that the amount of toxin produced must be taken into consideration. It is therefore of great importance to gain more knowledge about how the gene content of the replication region influences regulation of the phage life cycle and, consequently, the levels of Stx produced. More research is also needed to understand how different CI repressor types react to environmental stressors such as the host immune system and antibiotic treatment and the impact of these factors on the Stx production. Importantly, this work highlights that our understanding of bacterial pathogens cannot solely be based on studies on a few model bacterial strains and/or phage types.

## Conclusion

Some EHEC strains appear more virulent than others and increased knowledge on how characteristics of the replication region influence the level of Stx produced is important for understanding the mechanisms behind their pathogenicity. The present study revealed ten novel Eru types encoding phage replication proteins as well as a broad variation in the amino acid sequence of the CI repressor proteins which regulate the transcription of the replication proteins. This diversity has the potential to explain why certain EHEC strains are more pathogenic than others and the study forms an important knowledge platform for further investigations on how characteristics of the Stx phage genome influences the virulence of the host EHEC strain.

## Methods

A total of 120 Stx-converting phage genome sequences were retrieved from the NCBI virus database (taxid:10,239) by Standard Nucleotide BLAST using the A subunit of *stx1* (M19437.1) and *stx2* (AF125520) as query sequences (August 2021) (Additional file 1).

In addition, ten different bio-projects comprising European STEC strains, one Dutch (PRJNA285020), one Norwegian (PRJEB6447), one French (PRPRJNA706995), three Swiss (PRJNA680568, PRJNA694525, PRJNA438214), one English (PRJNA248042), one Italian (PRJNA666781), one German (PRJNA715185) and one Portuguese (PRJNA643688), were examined for contigs containing *stx* using BLAST as described above (Additional file 2). The dataset contained more than 3000 STEC isolates, however, the majority of contigs were too short (< 8000 bp) to contain *cI* and *stx* genes on the same contig thus only contigs larger than 8000 bp were examined. A total of 298 contigs containing the region between the CI-coding gene and the *stx* genes were identified in the dataset. The sequences were examined using pDRAW and Eru types were defined by the proteins encoded by the two genes located directly upstream of *cI*. GenomeNet Motif Search (Kyoto University Bioinformatics Center) was used for detection of protein motifs [54]. Erus were numbered consecutively as they were detected.

The 260 CI protein sequences (Additional file 3), mainly extracted from the abovementioned nucleotide sequences, were aligned using ClustalOmega [55]. A maximum likelihood tree was inferred from the alignment using IQ-TREE v1.6.12 [56]. Node supports were evaluated using the option -bb for ultrafast bootstraps [57] and the VT + GT model was selected as the best evolutionary model using ModelFinder and the BIC criterion [58]. Interactive Tree Of Life (iTOL) v6.4 was used for visualization [59].

## Supplementary Information

**Additional file 1.** Stx-converting phage genomes with Eru type

**Additional file 2.** BioProjects comprising European STEC strains with Eru type

**Additional file 3.** Information about the 260 CI sequences used in phylogenetic analysis

**Additional file 4.** Percent Identity Matrix (Clustal2.1) between clades of CI repressor sequences

## Data Availability

Data generated and analyzed throughout this study are included in this published article and in the additional information files. Stx-converting phage genome sequences were retrieved from the NCBI virus database (taxid:10,239) and each accession number can be found in Additional file 1. Accession number *stx* sequences from ten different bio-projects comprising European STEC strains are listed in Additional file 2. Accession number of protein sequences used for the phylogenetic analysis are listed in additional file 3.

## Abbreviations

AAA: ATPases Associated with diverse cellular Activities
BLAST: Basic Local Alignment Search Tool
DNA: Deoxyribonucleic acid
EHEC: Enterohaemorrhagic *E. coli*
Eru: EHEC phage replication units
HUS: Hemolytic uremic syndrome
HTH: Helix-turn-helix
NCBI: National Center for Biotechnology Information
STEC: Shiga toxin-producing *E. coli*
Stx: Shiga toxin
