# Supplementary material for: High diversity in the regulatory region of Shiga toxin encoding bacteriophages
Source: BMC Genomics. 2022 Mar 24;23:230. doi: 10.1186/s12864-022-08428-5 (PMC8951638; doi:10.1186/s12864-022-08428-5)
Supplement: Supplementary file 1 — Additional file 1. Stx-converting phage genomes with Eru type [file 12864_2022_8428_MOESM1_ESM.pdf]

Additional file 1

Stx-converting phage genomes with Eru type

Phage	Serotype	Acc no	Country	Year	Stx type	Eru type
Shigella phage 75/02 Stx	Shigella	NC_029120.1	Ungarn	2013	Stx1	Eru4
Shigella phage POCJ13	Shigella	KJ603229	USA	2014	Stx1	Eru4
Stx2 Converting phage I	O157:H7	AP004402	Japan	2001	Stx2a	lambdoid
Stx2 Converting phage II	O157:H7	AP005154	Japan	2002	Stx2	Eru3
Stx2 Converting phage 1717	O157:H7	FJ188381	Canada	2008	Stx2a	Eru2
Escherichia phage PA4	O157:H7	KP682372.1	USA	2015	Stx2a	Eru3
Escherichia phage PA5	O157:H7	KP682373.1	USA	2015	Stx2a	Eru3
Escherichia phage PA11	O157:H7	KP682375.1	USA	2015	Stx2a	Eru3
Escherichia phage PA12	O157:H7	KP682376.1	USA	2015	Stx2a	Eru3
Escherichia phage PA16	O157:H7	KP682377.1	USA	2015	Stx2a	Eru3
Escherichia phage PA21	O157:H7	KP682379.1	USA	2015	Stx2a	Eru3
Escherichia phage PA27	O157:H7	KP682380.1	USA	2015	Stx2a	Eru3
Escherichia phage PA28	O157:H7	KP682381.1	USA	2015	Stx2a	lambdoid
Escherichia phage PA29	O157:H7	KP682382.1	USA	2015	Stx2a	Eru3
Escherichia phage PA36	O157:H7	KP682386.1	USA	2015	Stx2a	Eru3
Escherichia phage PA42	O157:H7	KP682387.1	USA	2015	Stx2a	Eru3
Escherichia phage PA45	O157:H7	KP682389.1	USA	2015	Stx2a	Eru3
Escherichia phage PA50	O157:H7	KP682390.1	USA	2015	Stx2a	Eru3
Escherichia phage PA52	O157:H7	KP682392.1	USA	2015	Stx2a	Eru3
Escherichia phage P13771	O104:H4	HG792104.1	Germany	2009	Stx2a	Eru1
Escherichia phage P14437	O104:H4	HG792105.1	Norway	2006	Stx2a	Eru1
Escherichia phage P13363	O104:H4	HG803182.1	Germany	2011	Stx2a	Eru1
Escherichia phage P13374 proviral	O104:H4	HE664024.1	Germany	2011	Stx2a	Eru1
Enterobacteria phage YYZ-2008	O157:H7	FJ184280	Canada	2008	Stx1	Eru2
Escherichia phage SH2026Stx1	O157:H7	NC_049919.1	USA	2018	Stx1	Eru2
Escherichia phage GER2	O117:H7	MG710528.1	UK	2017	Stx1	Eru1
Escherichia Stx1-converting recombinant phage HUN/2013	O157:H7	KJ909655.1	Hungary	2013	Stx1	Eru3
Stx1 converting phage	O157:H7	AP005153.1	Japan	2003	Stx1	Eru3
Bacteriophage CP-1639 and chromosomal integration site	O111:H-	AJ304858.2	Germany	2000	Stx1	Eru3
Phage BP-4795 complete genome	O84:H4	AJ556162.1	Germany	2003	Stx1	lambdoid
Stx2-converting phage Stx2a_WGPS9 proviral	O157:H7	AP012535.1	Japan	2012	Stx2a	lambdoid
Stx2-converting phage Stx2a_F403 proviral	O157:H7	AP012529.1	Japan	2012	Stx2a	Eru5
Stx2-converting phage Stx2a_F422 proviral	O157:H7	AP012531.1	Japan	2012	Stx2a	lambdoid
Stx2-converting phage Stx2a_F451 proviral	O157:H7	AP012532.1	Japan	2012	Stx2a	Eru5
Stx2-converting phage Stx2a_F723 proviral	O157:H7	AP012533.1	Japan	2012	Stx2a	lambdoid
Escherichia phage P13803	O2:H27	HG792102.1	Germany	2013	Stx2a	Eru1

Stx2-converting phage Stx2a_F765 proviral	O157:H7	AP012534.1	Japan	2012	Stx2a	Eru1
Stx2-converting phage 86	O86:H-	NC_008464.1	USA	2003	Stx2a	Eru3
Stx2-converting phage Stx2a_WGPS8 proviral	O157:H7	AP012540.1	Japan	2012	Stx2c	Eru2
Stx2-converting phage Stx2a_WGPS4 proviral	O157:H7	AP012538.1	Japan	2012	Stx2c	Eru2
Stx2-converting phage Stx2a_F349 proviral	O157:H7	AP012530.1	Japan	2012	Stx2c	Eru2
Stx2-converting phage 1717, complete prophage genome	O157:H7	NC_011357.1	Canada	2008	Stx2c	Eru2
Enterobacteria phage 2851	O157:H7	FM180578.1	Germany	1993	Stx2c	Eru2
Stx2-converting phage Stx2a_WGPS6 proviral	O157:H7	AP012539.1	Japan	2012	Stx2c	Eru2
Stx2-converting phage Stx2a_WGPS2 proviral	O157:H7	AP012537.1	Japan	2012	Stx2c	Eru6
Stx2-converting phage Stx2a_1447 proviral	O157:H7	AP012536.1	Japan	2012	Stx2c	Eru6
Stx2 converting phage vB_EcoP_24B	O157:H7	HM208303.1	UK	2010	Stx2	Eru5
Enterobacteria phage Min27	O157:H7	NC_010237.1	China	2007	Stx2a	Eru5
Lys8385Vzw	O103:H11	MT225100	Japan	2020	Stx1	Eru6
Lys19259Vzw	O157:H7	MT225101	Japan	2020	Stx2	lambdoid
Bacteriophage P27	Ont:H-	AJ298298.1	Germany	2001	stx2e	Eru7
Stx1 converting phage AU5Stx1	O157	KU977419.1	Australia	2016	Stx1	lambdoid
Stx1 converting phage AU6Stx1	O157	KU977420.1	Australia	2016	Stx1	lambdoid
Stx2a-converting phage Stx2_14040	O145:H28	LC567818.1	Japan	2020	Stx2a	Eru7
Stx1a-converting phage Stx1_14040	O145:H28	LC567819.1	Japan	2020	Stx1	Eru7
Stx2a-converting phage Stx2_14744	O145:H28	LC567820.1	Japan	2020	Stx2a	Eru7
Stx1a-converting phage Stx1_14744	O145:H28	LC567821.1	Japan	2020	Stx1	Eru7
Stx1a-converting phage Stx1_132418	O145:H28	LC567823.1	Japan	2020	Stx1	Eru7
Stx2a-converting phage Stx2_499	O145:H28	LC567824.1	Japan	2020	Stx2a	Eru7
Stx1a-converting phage Stx1_499	O145:H28	LC567825.1	Japan	2020	Stx1	Eru7
Stx1a-converting phage Stx1_699	O145:H28	LC567827.1	Japan	2020	Stx1	Eru7
Stx2a-converting phage Stx2_EH2201	O145:H28	LC567829.1	Japan	2020	Stx2a	Eru7
Stx2d-converting phage Stx2_112808	O145:H28	LC567830.1	Japan	2020	Stx2a	Eru7
Stx1a-converting phage Stx1_EH1995	O145:H28	LC567831.1	Japan	2020	Stx1	Eru7
Stx1a-converting phage Stx1_EH1992	O145:H28	LC567833.1	Japan	2020	Stx1	Eru7
Stx2a-converting phage Stx2_EH1910	O145:H28	LC567834.1	Japan	2020	Stx2a	Eru7
Stx2a-converting phage Stx2_EH2246	O145:H28	LC567837.1	Japan	2020	Stx2a	Eru7
Stx2a-converting phage Stx2_95	O145:H28	LC567838.1	Japan	2020	Stx2a	Eru7
Stx2a-converting phage Stx2_EH1846	O145:H28	LC567840.1	Japan	2020	Stx2a	Eru7
Stx2a-converting phage Stx2_12E129_PPompW	O145:H28	LC567841.1	Japan	2020	Stx2a	Eru7
Stx2a-converting phage Stx2_12E129_yecE	O145:H28	LC567842.1	Japan	2020	Stx2a	Eru7
Stx2a-converting phage Stx2_EH0505	O121:H19	LC616031.1	Japan	2021	Stx2a	Eru2
Stx2a-converting phage Stx2_EH1965	O121:H19	LC616032.1	Japan	2021	Stx2a	Eru2
Stx2a-converting phage Stx2_EH0787	O121:H19	LC616033.1	Japan	2021	Stx2a	Eru2
Stx2a-converting phage Stx2_EH0337	O121:H19	LC616034.1	Japan	2021	Stx2a	Eru2
Stx2a-converting phage Stx2_13E027	O121:H19	LC616035.1	Japan	2021	Stx2a	Eru2
Stx2a-converting phage Stx2_PV06-102	O121:H19	LC616036.1	Japan	2021	Stx2a	Eru2

Stx2a-converting phage Stx2_4151	O121:H19	LC616037.1	Japan	2021	Stx2a	Eru2
Stx2a-converting phage Stx2_707	O121:H19	LC616038.1	Japan	2021	Stx2a	Eru2
Stx2a-converting phage Stx2_131033	O121:H19	LC616039.1	Japan	2021	Stx2a	Eru2
Stx2a-converting phage Stx2_3993	O121:H19	LC616040.1	Japan	2021	Stx2a	Eru2
Stx2a-converting phage Stx2_PV12-16	O121:H19	LC616041.1	Japan	2021	Stx2a	Eru2
Stx2a-converting phage Stx2_12E064	O121:H19	LC616042.1	Japan	2021	Stx2a	Eru2
Stx2a-converting phage Stx2_3725	O121:H19	LC616043.1	Japan	2021	Stx2a	Eru2
Stx2a-converting phage Stx2_8243	O121:H19	LC616044.1	Japan	2021	Stx2a	Eru2
Stx2a-converting phage Stx2_481	O121:H19	LC616045.1	Japan	2021	Stx2a	Eru2
Stx2a-converting phage Stx2_5122	O121:H19	LC616046.1	Japan	2021	Stx2a	Eru2
Stx2a-converting phage Stx2_6804	O121:H19	LC616047.1	Japan	2021	Stx2a	Eru2
Stx2a-converting phage Stx2_07Y06	O121:H19	LC616048.1	Japan	2021	Stx2a	Eru2
Stx2a-converting phage Stx2_11Y11	O121:H19	LC616049.1	Japan	2021	Stx2a	Eru2
Stx2a-converting phage Stx2_3350	O121:H19	LC616050.1	Japan	2021	Stx2a	Eru2
Stx2a-converting phage Stx2_PV07-173	O121:H19	LC616051.1	Japan	2021	Stx2a	Eru2
Stx2a-converting phage Stx2_7982	O121:H19	LC616052.1	Japan	2021	Stx2a	Eru2
Stx2a-converting phage Stx2_12849	O121:H19	LC616053.1	Japan	2021	Stx2a	Eru2
Stx2a-converting phage Stx2_12E092	O121:H19	LC616054.1	Japan	2021	Stx2a	Eru2
Stx2a-converting phage Stx2_1603	O121:H19	LC616055.1	Japan	2021	Stx2a	Eru2
Stx2a-converting phage Stx2_3772	O121:H19	LC616056.1	Japan	2021	Stx2a	Eru2
Stx2a-converting phage Stx2_579	O121:H19	LC616057.1	Japan	2021	Stx2a	Eru2
Stx2a-converting phage Stx2_716	O121:H19	LC616058.1	Japan	2021	Stx2a	Eru2
Stx2a-converting phage Stx2_3105	O121:H19	LC616059.1	Japan	2021	Stx2a	Eru2
Stx2a-converting phage Stx2_3417	O121:H19	LC616060.1	Japan	2021	Stx2a	Eru2
Stx2a-converting phage Stx2_12874	O121:H19	LC616061.1	Japan	2021	Stx2a	Eru2
Stx2a-converting phage Stx2_3896	O121:H19	LC616062.1	Japan	2021	Stx2a	Eru2
Stx2a-converting phage Stx2_3418	O121:H19	LC616063.1	Japan	2021	Stx2a	Eru2
Stx2a-converting phage Stx2_453	O121:H19	LC616064.1	Japan	2021	Stx2a	Eru2
Stx2a-converting phage Stx2_463	O121:H19	LC616065.1	Japan	2021	Stx2a	Eru2
Stx2a-converting phage Stx2_11106	O121:H19	LC616066.1	Japan	2021	Stx2a	Eru2
Stx2a-converting phage Stx2_356	O121:H19	LC616067.1	Japan	2021	Stx2a	Eru2
Stx2a-converting phage Stx2_522	O121:H19	LC616068.1	Japan	2021	Stx2a	Eru2
Stx2a-converting phage Stx2_635	O121:H19	LC616069.1	Japan	2021	Stx2a	Eru2
Stx2a-converting phage Stx2_15436	O121:H19	LC616070.1	Japan	2021	Stx2a	Eru2
Stx2a-converting phage Stx2_8048	O121:H19	LC616071.1	Japan	2021	Stx2a	Eru2
Stx2a-converting phage Stx2_PV03-14	O121:H19	LC616072.1	Japan	2021	Stx2a	Eru2
Stx2a-converting phage Stx2_08E027	O121:H19	LC616073.1	Japan	2021	Stx2a	Eru2
Stx2a-converting phage Stx2_10902	O121:H19	LC616074.1	Japan	2021	Stx2a	Eru2
Stx2a-converting phage Stx2_06E050	O121:H19	LC616075.1	Japan	2021	Stx2a	Eru2
Stx2a-converting phage Stx2_2935	O121:H19	LC616076.1	Japan	2021	Stx2a	Eru2
Stx2a-converting phage Stx2_13518	O121:H19	LC616077.1	Japan	2021	Stx2a	Eru2

Stx2a-converting phage Stx2_10E082	O121:H19	LC616078.1	Japan	2021	Stx2a	Eru2
Stx2a-converting phage Stx2_KH16-043	O121:H19	LC616079.1	Japan	2021	Stx2a	Eru7
