# Supplementary material for: High diversity in the regulatory region of Shiga toxin encoding bacteriophages
Source: BMC Genomics. 2022 Mar 24;23:230. doi: 10.1186/s12864-022-08428-5 (PMC8951638; doi:10.1186/s12864-022-08428-5)
Supplement: Supplementary file 2 — Additional file 2. BioProjects comprising European STEC strains with Eru type [file 12864_2022_8428_MOESM2_ESM.pdf]

Additional file 2

BioProjects comprising European STEC strains with Eru type

**PRJNA285020<br>Shiga toxin-producing *Escherichia coli* isolates obtained from two Dutch regions**		
**Strain**	**NCBI Accession no**	**Eru type**
**Stx2**		
Escherichia coli strain E09/10 contig_97, whole genome shotgun sequence	LGBK01000097.1	ND
Escherichia coli strain STEC 1109 contig_168, whole genome shotgun sequence	LGBC01000168.1	ND
Escherichia coli strain STEC 1255 STEC1255_contig_195, whole genome shotgun sequence	LOGC01000107.1	ND
Escherichia coli strain STEC 1293 STEC1293_contig_49, whole genome shotgun sequence	LOGF01000197.1	Eru7
Escherichia coli strain STEC 1299 STEC1299_contig_22, whole genome shotgun sequence	LOGG01000057.1	Eru6
Escherichia coli strain STEC 1363 STEC1363_contig_23, whole genome shotgun sequence	LOGI01000090.1	Eru6
Escherichia coli strain STEC 1375 STEC1375_contig_25, whole genome shotgun sequence	LOGJ01000018.1	Eru7
Escherichia coli strain STEC 1442 STEC1442_contig_2, whole genome shotgun sequence	LOGK01000083.1	Eru7
Escherichia coli strain STEC 1506 STEC1506_contig_10, whole genome shotgun sequence	LPWV01000002.1	ND
Escherichia coli strain STEC 1528 STEC1528_contig_43, whole genome shotgun sequence	LOGP01000038.1	ND
Escherichia coli strain STEC 1634 STEC1634_contig_43, whole genome shotgun sequence	LOGS01000097.1	ND
Escherichia coli strain STEC 1686 STEC1686_contig_77, whole genome shotgun sequence	LOGT01000177.1	Eru10
Escherichia coli strain STEC 200 STEC-200_contig_39, whole genome shotgun sequence	LNZL01000033.1	Eru7
Escherichia coli strain STEC 2075 contig_86, whole genome shotgun sequence	LGBD01000086.1	ND
Escherichia coli strain STEC 2110.1 2110-1_contig_200, whole genome shotgun sequence	LPWW01000114.1	ND
Escherichia coli strain STEC 2110.1 2110-1_contig_204, whole genome shotgun sequence	LPWW01000118.1	ND
Escherichia coli strain STEC 2110.1 2110-1_contig_232, whole genome shotgun sequence	LPWW01000149.1	ND
Escherichia coli strain STEC 2110.1 2110-1_contig_40, whole genome shotgun sequence	LPWW01000216.1	ND
Escherichia coli strain STEC 2112 contig_150, whole genome shotgun sequence	LGBE01000150.1	ND
Escherichia coli strain STEC 2236 STEC2236_contig_23, whole genome shotgun sequence	LOGY01000073.1	lambdoid
Escherichia coli strain STEC 2257 contig_109, whole genome shotgun sequence	LGBF01000109.1	ND
Escherichia coli strain STEC 2410 contig_189, whole genome shotgun sequence	LGBG01000189.1	ND
Escherichia coli strain STEC 2410 contig_203, whole genome shotgun sequence	LGBG01000203.1	ND
Escherichia coli strain STEC 2410 contig_72, whole genome shotgun sequence	LGBG01000072.1	Eru1
Escherichia coli strain STEC 2450 STEC2450_contig_65, whole genome shotgun sequence	LPXA01000177.1	Eru6
Escherichia coli strain STEC 2499 STEC2499_contig_42, whole genome shotgun sequence	LOIE01000070.1	ND
Escherichia coli strain STEC 2505 STEC2505_contig_24, whole genome shotgun sequence	LPXB01000100.1	Eru6
Escherichia coli strain STEC 2573 STEC2573_contig_104, whole genome shotgun sequence	LOIH01000007.1	ND
Escherichia coli strain STEC 2573 STEC2573_contig_138, whole genome shotgun sequence	LOIH01000044.1	ND
Escherichia coli strain STEC 2573 STEC2573_contig_62, whole genome shotgun sequence	LOIH01000107.1	ND
Escherichia coli strain STEC 2573 STEC2573_contig_7, whole genome shotgun sequence	LOIH01000115.1	ND
Escherichia coli strain STEC 2591 STEC2591_contig_56, whole genome shotgun sequence	LOII01000068.1	ND
Escherichia coli strain STEC 2595 STEC2595_contig_34, whole genome shotgun sequence	LOIJ01000033.1	Eru11

Escherichia coli strain STEC 2620 STEC2620_contig_169, whole genome shotgun sequence	LPXC01000078.1	ND
Escherichia coli strain STEC 2620 STEC2620_contig_226, whole genome shotgun sequence	LPXC01000142.1	ND
Escherichia coli strain STEC 2620 STEC2620_contig_69, whole genome shotgun sequence	LPXC01000201.1	ND
Escherichia coli strain STEC 2667 contig_19, whole genome shotgun sequence	LGBH01000019.1	ND
Escherichia coli strain STEC 2746 STEC2746_contig_61, whole genome shotgun sequence	LPXD01000148.1	Eru6
Escherichia coli strain STEC 2770 STEC2770_contig_18, whole genome shotgun sequence	LPXF01000088.1	ND
Escherichia coli strain STEC 2797 STEC2797_contig_85, whole genome shotgun sequence	LOIP01000113.1	ND
Escherichia coli strain STEC 2820 contig_161, whole genome shotgun sequence	LGBQ01000161.1	ND
Escherichia coli strain STEC 2821 contig_98, whole genome shotgun sequence	LGBI01000098.1	ND
Escherichia coli strain STEC 2826 STEC2826_contig_97, whole genome shotgun sequence	LOJA01000134.1	ND
Escherichia coli strain STEC 2861 STEC2861_contig_8, whole genome shotgun sequence	LOIR01000058.1	Eru11
Escherichia coli strain STEC 2868 contig_83, whole genome shotgun sequence	LGBJ01000083.1	ND
Escherichia coli strain STEC 2894.2 2894-2_contig_216, whole genome shotgun sequence	LOIT01000131.1	ND
Escherichia coli strain STEC 2953 STEC2953_contig_5, whole genome shotgun sequence	LOIW01000084.1	Eru10
Escherichia coli strain STEC 2954 STEC2954_contig_124, whole genome shotgun sequence	LPXE01000029.1	ND
Escherichia coli strain STEC 2980 STEC2980_contig_28, whole genome shotgun sequence	LOIY01000123.1	Eru6
Escherichia coli strain STEC 3031 STEC3031_contig_25, whole genome shotgun sequence	LOIZ01000129.1	Eru6
Escherichia coli strain STEC 3039 STEC3039_contig_118, whole genome shotgun sequence	LPUH01000022.1	Eru6
Escherichia coli strain STEC 3039 STEC3039_contig_29, whole genome shotgun sequence	LPUH01000108.1	Eru6
Escherichia coli strain STEC 3055 STEC3055_contig_130, whole genome shotgun sequence	LPUI01000036.1	ND
Escherichia coli strain STEC 3055 STEC3055_contig_149, whole genome shotgun sequence	LPUI01000056.1	ND
Escherichia coli strain STEC 3055 STEC3055_contig_201, whole genome shotgun sequence	LPUI01000115.1	ND
Escherichia coli strain STEC 3098 STEC3098_contig_22, whole genome shotgun sequence	LPUM01000015.1	Eru6
Escherichia coli strain STEC 343 contig_50, whole genome shotgun sequence	LDOZ01000050.1	ND
Escherichia coli strain STEC 440 440_contig_159, whole genome shotgun sequence	MRVY01000159.1	ND
Escherichia coli strain STEC 476-14 476-14_contig_49, whole genome shotgun sequence	MRVU01000049.1	Eru7
Escherichia coli strain STEC 479BS2 479BS2_contig_11, whole genome shotgun sequence	MRVR01000011.1	Eru7
Escherichia coli strain STEC 480-3 480-3_contig_132, whole genome shotgun sequence	MRVT01000132.1	ND
Escherichia coli strain STEC 510-5 510-5_contig_40, whole genome shotgun sequence	MRVV01000040.1	Eru7
Escherichia coli strain STEC 514-2 514-2_contig_122, whole genome shotgun sequence	MRVZ01000122.1	Eru7
Escherichia coli strain STEC 536-9 Re_536-9_contig_20, whole genome shotgun sequence	MRVS01000020.1	Eru7
Escherichia coli strain STEC 545 STEC-545_contig_46, whole genome shotgun sequence	LODB01000401.1	Eru8
Escherichia coli strain STEC 563 STEC-563_contig_138, whole genome shotgun sequence	LODD01000044.1	ND
Escherichia coli strain STEC 563 STEC-563_contig_148, whole genome shotgun sequence	LODD01000055.1	ND
Escherichia coli strain STEC 565 STEC565_contig_23, whole genome shotgun sequence	LODE01000106.1	Eru2
Escherichia coli strain STEC 573-4 573-4_contig_15, whole genome shotgun sequence	MRWA01000015.1	Eru7
Escherichia coli strain STEC 587-5 587-5_contig_81, whole genome shotgun sequence	MRWB01000081.1	ND
Escherichia coli strain STEC 587-5 587-5_contig_82, whole genome shotgun sequence	MRWB01000082.1	ND
Escherichia coli strain STEC 605 STEC-605_contig_136, whole genome shotgun sequence	LFUA01000136.1	ND
Escherichia coli strain STEC 621S1 621S1_contig_13, whole genome shotgun sequence	MRVX01000013.1	ND
Escherichia coli strain STEC 623 STEC-623_contig_109, whole genome shotgun sequence	LFUB01000109.1	ND

Escherichia coli strain STEC 625C-4 625C-4_contig_16, whole genome shotgun sequence	MRVW01000016.1	Eru7
Escherichia coli strain STEC 66 STEC-66_contig_79, whole genome shotgun sequence	LNFT01000172.1	Eru1
Escherichia coli strain STEC 709 STEC-709_contig_1, whole genome shotgun sequence	LOFM01000001.1	Eru1
Escherichia coli strain STEC 709 STEC-709_contig_121, whole genome shotgun sequence	LOFM01000026.1	Eru7
Escherichia coli strain STEC 731 STEC-731_contig_50, whole genome shotgun sequence	LOFN01000117.1	Eru7
Escherichia coli strain STEC 771 contig_171, whole genome shotgun sequence	LGAZ01000171.1	ND
Escherichia coli strain STEC 915 STEC-915_contig_74, whole genome shotgun sequence	LFUH01000074.1	ND
Escherichia coli strain STEC 931 STEC-931_contig_69, whole genome shotgun sequence	LOFS01000183.1	Eru1
Escherichia coli strain STEC 989 contig_159, whole genome shotgun sequence	LGBA01000159.1	ND
Escherichia coli strain STEC 994 contig_120, whole genome shotgun sequence	LGBB01000120.1	ND
**Stx1**		
Escherichia coli strain STEC 1117 STEC1117_contig_150, whole genome shotgun sequence	LOFU01000058.1	ND
Escherichia coli strain STEC 1161 STEC1161_contig_128, whole genome shotgun sequence	LOFV01000033.1	ND
Escherichia coli strain STEC 1188 STEC1188_contig_25, whole genome shotgun sequence	LOFX01000035.1	Eru4
Escherichia coli strain STEC 1201 STEC1201_contig_80, whole genome shotgun sequence	LOFZ01000160.1	ND
Escherichia coli strain STEC 1225 STEC1225_contig_97, whole genome shotgun sequence	LOGA01000204.1	ND
Escherichia coli strain STEC 1236 STEC1236_contig_228, whole genome shotgun sequence	LOGB01000144.1	ND
Escherichia coli strain STEC 1255 STEC1255_contig_18, whole genome shotgun sequence	LOGC01000090.1	ND
Escherichia coli strain STEC 1284 STEC1284_contig_140, whole genome shotgun sequence	LOGE01000047.1	ND
Escherichia coli strain STEC 1293 STEC1293_contig_188, whole genome shotgun sequence	LOGF01000099.1	ND
Escherichia coli strain STEC 1299 STEC1299_contig_7, whole genome shotgun sequence	LOGG01000109.1	Eru4
Escherichia coli strain STEC 1465 STEC1465_contig_2, whole genome shotgun sequence	LOGL01000097.1	Eru1
Escherichia coli strain STEC 1500 STEC1500_contig_18, whole genome shotgun sequence	LOGN01000031.1	Eru4
Escherichia coli strain STEC 1506 STEC1506_contig_62, whole genome shotgun sequence	LPWV01000121.1	Eru4
Escherichia coli strain STEC 1513 STEC1513_contig_128, whole genome shotgun sequence	LOGO01000033.1	ND
Escherichia coli strain STEC 1532 STEC1532_contig_89, whole genome shotgun sequence	LOGQ01000160.1	lambdoid
Escherichia coli strain STEC 1585 STEC1585_contig_10, whole genome shotgun sequence	LOGR01000002.1	Eru4
Escherichia coli strain STEC 168 STEC-168_contig_23, whole genome shotgun sequence	LNFV01000026.1	Eru4
Escherichia coli strain STEC 1686 STEC1686_contig_68, whole genome shotgun sequence	LOGT01000167.1	ND
Escherichia coli strain STEC 169 STEC169_contig_145, whole genome shotgun sequence	LNZJ01000052.1	ND
Escherichia coli strain STEC 196 STEC196_contig_6, whole genome shotgun sequence	LNZK01000067.1	Eru4
Escherichia coli strain STEC 2064 STEC2064_contig_11, whole genome shotgun sequence	LOJC01000003.1	Eru4
Escherichia coli strain STEC 2074 STEC2074_contig_35, whole genome shotgun sequence	LOJD01000082.1	Eru4
Escherichia coli strain STEC 2112 contig_161, whole genome shotgun sequence	LGBE01000161.1	ND
Escherichia coli strain STEC 2144 STEC2144_contig_132, whole genome shotgun sequence	LOGU01000038.1	lambdoid
Escherichia coli strain STEC 2174 STEC2174_contig_4, whole genome shotgun sequence	LOGV01000046.1	Eru4
Escherichia coli strain STEC 2193 STEC2193_contig_13, whole genome shotgun sequence	LOGW01000035.1	lambdoid
Escherichia coli strain STEC 2211 STEC2211_contig_137, whole genome shotgun sequence	LOGX01000043.1	ND
Escherichia coli strain STEC 2257 contig_143, whole genome shotgun sequence	LGBF01000143.1	ND
Escherichia coli strain STEC 2270 STEC2270_contig_37, whole genome shotgun sequence	LPWX01000147.1	ND
Escherichia coli strain STEC 2346 STEC2346_contig_159, whole genome shotgun sequence	LOHA01000067.1	ND

Escherichia coli strain STEC 2359 STEC2359_contig_30, whole genome shotgun sequence	LOHB01000024.1	ND
Escherichia coli strain STEC 2363 STEC2363_contig_20, whole genome shotgun sequence	LPWY01000046.1	Eru4
Escherichia coli strain STEC 2419 STEC2419_contig_65, whole genome shotgun sequence	LPWZ01000120.1	lambdoid
Escherichia coli strain STEC 2441 STEC2441_contig_165, whole genome shotgun sequence	LOHC01000074.1	ND
Escherichia coli strain STEC 2450 STEC2450_contig_19, whole genome shotgun sequence	LPXA01000101.1	Eru4
Escherichia coli strain STEC 2499 STEC2499_contig_39, whole genome shotgun sequence	LOIE01000066.1	Eru7
Escherichia coli strain STEC 2505 STEC2505_contig_22, whole genome shotgun sequence	LPXB01000098.1	Eru4
Escherichia coli strain STEC 2564 STEC2564_contig_44, whole genome shotgun sequence	LOIG01000176.1	Eru1
Escherichia coli strain STEC 2573 STEC2573_contig_61, whole genome shotgun sequence	LOIH01000106.1	ND
Escherichia coli strain STEC 2591 STEC2591_contig_23, whole genome shotgun sequence	LOII01000032.1	Eru7
Escherichia coli strain STEC 2620 STEC2620_contig_99, whole genome shotgun sequence	LPXC01000234.1	ND
Escherichia coli strain STEC 2633 STEC2633_contig_39, whole genome shotgun sequence	LOIK01000066.1	Eru5
Escherichia coli strain STEC 2667 contig_141, whole genome shotgun sequence	LGBH01000141.1	ND
Escherichia coli strain STEC 2708 STEC2708_contig_138, whole genome shotgun sequence	LOIL01000044.1	ND
Escherichia coli strain STEC 2743 STEC2743_contig_90, whole genome shotgun sequence	LOIM01000198.1	ND
Escherichia coli strain STEC 2746 STEC2746_contig_26, whole genome shotgun sequence	LPXD01000109.1	Eru4
Escherichia coli strain STEC 2764 STEC2764_contig_63, whole genome shotgun sequence	LOIN01000127.1	Eru4
Escherichia coli strain STEC 2770 STEC2770_contig_19, whole genome shotgun sequence	LPXF01000089.1	ND
Escherichia coli strain STEC 2788 STEC2788_contig_39, whole genome shotgun sequence	LOIO01000033.1	Eru4
Escherichia coli strain STEC 2820 contig_76, whole genome shotgun sequence	LGBQ01000076.1	ND
Escherichia coli strain STEC 2821 contig_145, whole genome shotgun sequence	LGBI01000145.1	ND
Escherichia coli strain STEC 2839 STEC2839_contig_22, whole genome shotgun sequence	LOJB01000015.1	Eru4
Escherichia coli strain STEC 2841 STEC2841_contig_11, whole genome shotgun sequence	LOIQ01000003.1	Eru4
Escherichia coli strain STEC 2868 contig_84, whole genome shotgun sequence	LGBJ01000084.1	ND
Escherichia coli strain STEC 2894.1 2894-1_contig_38, whole genome shotgun sequence	LOIS01000032.1	Eru5
Escherichia coli strain STEC 29 STEC-29_contig_6, whole genome shotgun sequence	LNFU01000056.1	Eru4
Escherichia coli strain STEC 2920 STEC2920_contig_172, whole genome shotgun sequence	LOIU01000082.1	ND
Escherichia coli strain STEC 2938 STEC2938_contig_47, whole genome shotgun sequence	LOIV01000111.1	ND
Escherichia coli strain STEC 2953 STEC2953_contig_44, whole genome shotgun sequence	LOIW01000078.1	Eru4
Escherichia coli strain STEC 2954 STEC2954_contig_9, whole genome shotgun sequence	LPXE01000152.1	Eru4
Escherichia coli strain STEC 2962 STEC2962_contig_4, whole genome shotgun sequence	LOIX01000034.1	Eru4
Escherichia coli strain STEC 2980 STEC2980_contig_92, whole genome shotgun sequence	LOIY01000194.1	Eru4
Escherichia coli strain STEC 299 STEC299_contig_145, whole genome shotgun sequence	LOCR01000052.1	ND
Escherichia coli strain STEC 3031 STEC3031_contig_24, whole genome shotgun sequence	LOIZ01000128.1	Eru4
Escherichia coli strain STEC 3039 STEC3039_contig_100, whole genome shotgun sequence	LPUH01000003.1	ND
Escherichia coli strain STEC 3055 STEC3055_contig_65, whole genome shotgun sequence	LPUI01000214.1	Eru1
Escherichia coli strain STEC 3084 STEC3084_contig_28, whole genome shotgun sequence	LPUJ01000040.1	Eru4
Escherichia coli strain STEC 3087 STEC3087_contig_27, whole genome shotgun sequence	LPUK01000020.1	Eru4
Escherichia coli strain STEC 3094 STEC3094_contig_88, whole genome shotgun sequence	LPUL01000162.1	ND
Escherichia coli strain STEC 3106 STEC3106_contig_9, whole genome shotgun sequence	LPUN01000125.1	Eru4
Escherichia coli strain STEC 329 STEC329_contig_68, whole genome shotgun sequence	LOCT01000103.1	Eru4

Escherichia coli strain STEC 370 STEC370_contig_124, whole genome shotgun sequence	LOCU01000029.1	ND
Escherichia coli strain STEC 380 STEC380_contig_200, whole genome shotgun sequence	LOCV01000114.1	ND
Escherichia coli strain STEC 384 STEC384_contig_129, whole genome shotgun sequence	LOCW01000034.1	ND
Escherichia coli strain STEC 440 440_contig_83, whole genome shotgun sequence	MRVY01000083.1	ND
Escherichia coli strain STEC 464 STEC464_contig_70, whole genome shotgun sequence	LOCX01000131.1	ND
Escherichia coli strain STEC 477 STEC477_contig_83, whole genome shotgun sequence	LOCY01000237.1	ND
Escherichia coli strain STEC 479 STEC479_contig_147, whole genome shotgun sequence	LOCZ01000054.1	ND
Escherichia coli strain STEC 487 STEC487_contig_273, whole genome shotgun sequence	LODA01000194.1	ND
Escherichia coli strain STEC 559 STEC559_contig_57, whole genome shotgun sequence	LODC01000145.1	lambdoid
Escherichia coli strain STEC 563 STEC-563_contig_155, whole genome shotgun sequence	LODD01000063.1	ND
Escherichia coli strain STEC 621S1 621S1_contig_12, whole genome shotgun sequence	MRVX01000012.1	ND
Escherichia coli strain STEC 623 STEC-623_contig_46, whole genome shotgun sequence	LFUB01000046.1	ND
Escherichia coli strain STEC 627 STEC627_contig_98, whole genome shotgun sequence	LODF01000184.1	ND
Escherichia coli strain STEC 645 STEC645_contig_5, whole genome shotgun sequence	LODG01000045.1	Eru4
Escherichia coli strain STEC 66 STEC-66_contig_78, whole genome shotgun sequence	LNFT01000171.1	ND
Escherichia coli strain STEC 690 STEC690_contig_253, whole genome shotgun sequence	LOFJ01000172.1	ND
Escherichia coli strain STEC 691 STEC691_contig_122, whole genome shotgun sequence	LOFK01000027.1	ND
Escherichia coli strain STEC 707 STEC707_contig_88, whole genome shotgun sequence	LOFL01000143.1	ND
Escherichia coli strain STEC 709 STEC-709_contig_147, whole genome shotgun sequence	LOFM01000054.1	ND
Escherichia coli strain STEC 757 STEC757_contig_136, whole genome shotgun sequence	LOFO01000042.1	ND
Escherichia coli strain STEC 764 STEC764_contig_242, whole genome shotgun sequence	LOFP01000160.1	ND
Escherichia coli strain STEC 771 contig_117, whole genome shotgun sequence	LGAZ01000117.1	ND
Escherichia coli strain STEC 886 STEC886_contig_19, whole genome shotgun sequence	LOFR01000041.1	Eru4
Escherichia coli strain STEC 915 STEC-915_contig_344, whole genome shotgun sequence	LFUH01000344.1	ND
Escherichia coli strain STEC 989 contig_82, whole genome shotgun sequence	LGBA01000082.1	ND
**PRJEB6447 Norwegian STEC isolates**		
**Strain**	**NCBI Accession no**	**Eru type**
**Stx2**		
FHI3	CCPT01000144.1	Eru7
FHI4	LM995944.1	Eru7
FHI5	CCQC01000098.1	ND
FHI6	LM996356.1	Eru1
FHI7	LM996408.1	Eru1
FHI9	LM997071.1	Eru1
FHI12	LM995551.1	Eru1
FHI19	CCPR01000384.1	Eru1
FHI22	CCPS01000056.1	ND
FHI24	LM995744.1	Eru1
FHI25	LM996297.1	Eru7
FHI27	LM995668.1	Eru1

FHI28	LM995766.1	ND
FHI30	LM995896.1	Eru10
FHI31	CCVO01000012.1	ND
FHI32	CCVP01000063.1	ND
FHI35	CCQJ01000092.1	ND
FHI36	CCPV01000170.1	Eru1
FHI37	CCPU01000077.1	ND
FHI39	CCPX01000131.1	Eru6
FHI41	CCPY01000042.1	lambdoid
FHI42	LK999941.1	ND
FHI43	LM996066.1	Eru7
FHI48	LM996025.1	Eru7 (HUS)
FHI49	CCQB01000137.1	ND
FHI53	CCRA01000038.1	Eru6
FHI58	LM995979.1	ND (HUS)
FHI59	LK999983.1	Eru6
FHI62	LN554923.1	Eru7
FHI63	LM996460.1	Eru7 (HUS)
FHI65	LM996529.1	Eru6
FHI71	LM996832.1	Eru6
FHI79	LM996682.1	ND (HUS)
FHI8	LM996720.1	Eru1 (HUS)
FHI81	CCQV01000126.1	ND
FHI82	LM996798.1	Eru7
FHI83	LM996896.1	Eru2 (HUS)
FHI85	LM996947.1	Eru1
FHI86	CCRC01000097.1	Eru1
FHI88	CCQX01000142.1	ND
FHI89	LM997036.1	Eru6
FHI92	LM997161.1	Eru7
FHI95	CCRD01000099.1	Eru7
FHI98	LM997367.1	Eru10
FHI99	LM997254.1	Eru13
FHI100	LK985420.1	Eru8
FHI101	CCPP01000171.1	Eru7
FHI102	LM995495.1	Eru1 (HUS)
StOlav104	LK931573.1	Eru7
St. Olav164	PVRW01000040.1	Eru7
**Stx1**		
FHI1	CCPO01000158.1	ND

FHI5	CCQC01000109.1	ND
FHI6	LM996340.1	ND (HUS)
FHI7	LM996413.1	Eru4 (HUS)
FHI20	LM995817.1	ND
FHI22	CCPS01000094.1	Eru4
FHI23	NZ_LM995659.1	Eru12
FHI29	LM995865.1	Eru4
FHI30	LM995905.1	Eru4
FHI32	CCVP01000073.1	Eru12
FHI34	LM996489.1	Eru4
FHI37	CCPU01000123.1	ND
FHI40	LM996246.1	ND
FHI45	CCQM01000063.1	Eru4
FHI46	CCPZ01000238.1	ND
FHI47	CCQA01000072.1	Eru6
FHI49	CCQB01000089.1	Eru4
FHI50	CCQD01000134.1	Eru6
FHI51	CCQW01000128.1	ND
FHI52	CCQH01000128.1	ND
FHI54	CCQI01000086.1	ND
FHI56	CCQK01000110.1	ND
FHI60	CCRB01000131.1	ND
FHI61	CCQL01000336.1	ND
FHI64	CCQN01000148.1	ND
FHI65	LM996514.1	Eru1
FHI67	CCQO01000029.1	ND
FHI68	CCQQ01000131.1	Eru1
FHI69	CCQP01000089.1	Eru4
FHI70	LM997107.1	Eru2
FHI72	LM996870.1	Eru1
FHI74	LM996604.1	Eru5
FHI75	LM996630.1	ND
FHI76	CCQT01000162.1	ND
FHI77	CCQS01000108.1	ND
FHI78	CCQU01000036.1	ND
FHI81	CCQV01000151.1	Eru1
FHI84	CCVQ01000033.1	ND
FHI85	LM996969.1	ND
FHI87	LM996984.1	ND
FHI90	LM997295.1	ND
FHI92	LM997172.1	Eru4

FHI93	CCQY01000267.1	ND
FHI96	CCRE01000139.1	Eru4
FHI97	LM997224.1	lambdoid
St. Olav143	CCVR01000362.1	ND
**PRJNA706995**<br>**Shiga-toxin-producing *Escherichia coli* carriage in asymptomatic French infants**		
**Strain**	**NCBI Accession no**	**Eru type**
**Stx2**		
Escherichia coli strain 172124GE NODE_96_259616.007695, whole genome shotgun sequence	JAFMZR010000326.1	ND
Escherichia coli strain 192068CE NODE_18_9463935.066618, whole genome shotgun sequence	JAFMZX010000018.1	Eru7
Escherichia coli strain 182303EI NODE_70377111.543908, whole genome shotgun sequence	JAFMZU010000233.1	ND
Escherichia coli strain 172332DM NODE_73_403113.378842, whole genome shotgun sequence	JAFMZS010000271.1	ND
Escherichia coli strain 182769NA NODE_67_1551313.870207, whole genome shotgun sequence	JAFMZW010000381.1	Eru7
Escherichia coli strain 172669GA NODE_53_1898931.577163, whole genome shotgun sequence	JAFMZT010000053.1	Eru6
**Stx1**		
Escherichia coli strain 192068CE NODE_23_7894751.250875, whole genome shotgun sequence	JAFMZX010000023.1	lambdoid
Escherichia coli strain 192161DA NODE_83_706311.409746, whole genome shotgun sequence	JAFMZY010000402.1	ND
Escherichia coli strain 182629BE NODE_112344714.641566, whole genome shotgun sequence	JAFMZV010000014.1	ND
**PRJNA694525**<br>**STEC isolated from raw meat-based diets for companion animals in Switzerland**		
**Strain**	**NCBI Accession no**	**Eru type**
**Stx2**		
Escherichia coli strain ATB6-118 contig00027, whole genome shotgun sequence	JAEUYO010000027.1	ND
Escherichia coli strain LSC6-3 contig00094, whole genome shotgun sequence	JAETZC010000094.1	ND
Escherichia coli strain ATC45-11 contig00118, whole genome shotgun sequence	JAETYS010000118.1	ND
Escherichia coli strain ATC46-2 contig00088, whole genome shotgun sequence	JAETYT010000088.1	ND
Escherichia coli strain ATC-9-6 contig00064, whole genome shotgun sequence	JAETYO010000064.1	ND
Escherichia coli strain ATC-44-40 contig00081, whole genome shotgun sequence	JAETYM010000081.1	ND
Escherichia coli strain ATB47-1 contig00030, whole genome shotgun sequence	JAETYG010000030.1	lambdoid
Escherichia coli strain ATB-15-29 contig00069, whole genome shotgun sequence	JAETYB010000069.1	ND
Escherichia coli strain LSB-2-42b contig00018, whole genome shotgun sequence	JAETYW010000018.1	Eru8
Escherichia coli strain LSB-2-27b contig00019, whole genome shotgun sequence	JAETYV010000019.1	Eru8
Escherichia coli strain ATC-B-20-47 contig00012, whole genome shotgun sequence	JAETYP010000012.1	Eru1
Escherichia coli strain ATC-21-17 contig00069, whole genome shotgun sequence	JAETYJ010000069.1	ND
Escherichia coli strain ATC-11-10 contig00077, whole genome shotgun sequence	JAETYH010000077.1	ND
Escherichia coli strain ATB-21-84 contig00067, whole genome shotgun sequence	JAETYC010000067.1	ND
Escherichia coli strain ATC39-3 contig00011, whole genome shotgun sequence	JAETYR010000011.1	ND
Escherichia coli strain ATB-39-11 contig00011, whole genome shotgun sequence	JAETYE010000011.1	ND
Escherichia coli strain ATB-11-12 contig00046, whole genome shotgun sequence	JAETXZ010000046.1	Eru10

Escherichia coli strain LSC6-3 contig00029, whole genome shotgun sequence	JAETZC010000029.1	Eru10
Escherichia coli strain ATB-23-31c contig00161, whole genome shotgun sequence	JAETYD010000161.1	ND
Escherichia coli strain ATB-10-31 contig00098, whole genome shotgun sequence	JAETXY010000098.1	ND
Escherichia coli strain LSC1-58 contig00138, whole genome shotgun sequence	JAETYZ010000138.1	ND
Escherichia coli strain ATC7-7 contig00075, whole genome shotgun sequence	JAETYU010000075.1	ND
Escherichia coli strain ATC-41-3 contig00056, whole genome shotgun sequence	JAETYL010000056.1	Eru6
Escherichia coli strain ATC-49-13 contig00211, whole genome shotgun sequence	JAETYN010000211.1	ND
Escherichia coli strain ATB29-1 contig00211, whole genome shotgun sequence	JAETYF010000211.1	ND
Escherichia coli strain LSC1-P21-24 contig00207, whole genome shotgun sequence	JAETZB010000207.1	ND
Escherichia coli strain LSB-P21-51 contig00208, whole genome shotgun sequence	JAETYX010000208.1	ND
Escherichia coli strain LSC1-P21-24 contig00170, whole genome shotgun sequence	JAETZB010000170.1	ND
Escherichia coli strain LSC1-P21-24 contig00095, whole genome shotgun sequence	JAETZB010000095.1	ND
Escherichia coli strain LSB-P21-51 contig00187, whole genome shotgun sequence	JAETYX010000187.1	ND
Escherichia coli strain ATB29-1 contig00181, whole genome shotgun sequence	JAETYF010000181.1	ND
Escherichia coli strain LSB-P21-51 contig00243, whole genome shotgun sequence	JAETYX010000243.1	ND
Escherichia coli strain ATB29-1 contig00102, whole genome shotgun sequence	JAETYF010000102.1	ND
Escherichia coli strain ATC-49-13 contig00089, whole genome shotgun sequence	JAETYN010000089.1	ND
**Stx1**		
Escherichia coli strain ATB6-118 contig00001, whole genome shotgun sequence	JAEUYO010000001.1	Eru4
Escherichia coli strain LSC1-P21-24 contig00106, whole genome shotgun sequence	JAETZB010000106.1	ND
Escherichia coli strain LSB-P21-51 contig00108, whole genome shotgun sequence	JAETYX010000108.1	ND
Escherichia coli strain ATC-4-67 contig00017, whole genome shotgun sequence	JAETYK010000017.1	Eru1
Escherichia coli strain ATC-15-17 contig00026, whole genome shotgun sequence	JAETYI010000026.1	Eru1
Escherichia coli strain ATB29-1 contig00088, whole genome shotgun sequence	JAETYF010000088.1	ND
Escherichia coli strain ATB-23-31c contig00074, whole genome shotgun sequence	JAETYD010000074.1	ND
Escherichia coli strain ATC45-11 contig00091, whole genome shotgun sequence	JAETYS010000091.1	ND
Escherichia coli strain LSC1-7 contig00009, whole genome shotgun sequence	JAETZA010000009.1	Eru4
Escherichia coli strain LSC1-58 contig00032, whole genome shotgun sequence	JAETYZ010000032.1	Eru4
Escherichia coli strain LSC-5-20 contig00002, whole genome shotgun sequence	JAETYY010000002.1	Eru4
Escherichia coli strain ATC7-7 contig00018, whole genome shotgun sequence	JAETYU010000018.1	Eru4
Escherichia coli strain ATC36-6 contig00045, whole genome shotgun sequence	JAETYQ010000045.1	Eru4
Escherichia coli strain ATB-14-66 contig00007, whole genome shotgun sequence	JAETYA010000007.1	Eru4
Escherichia coli strain ATB-10-31 contig00022, whole genome shotgun sequence	JAETXY010000022.1	Eru4
Escherichia coli strain ATB-23-31c contig00152, whole genome shotgun sequence	JAETYD010000152.1	ND
Escherichia coli strain ATB-23-31c contig00037, whole genome shotgun sequence	JAETYD010000037.1	

PRJNA680568
***Escherichia coli* O80:H2 strains from Switzerland**

Strain	NCBI Accession no	Eru type
**Stx2**		
Escherichia coli strain 1970-08 1970-08_contig00121, whole genome shotgun sequence	JAEANL010000121.1	ND
Escherichia coli strain S18-215 S18-215_contig00116, whole genome shotgun sequence	JAEANI010000116.1	ND

Escherichia coli strain S19-18-1 S19-18_contig00081, whole genome shotgun sequence	JAEANE010000081.1	Eru7
Escherichia coli strain S19-2-2 S19-2_contig00118, whole genome shotgun sequence	JAEAND010000118.1	ND
Escherichia coli strain S19-64-1 S19-64_contig00113, whole genome shotgun sequence	JAEANA010000113.1	ND
Escherichia coli strain S19-677-1 S19-677_contig00129, whole genome shotgun sequence	JAEAMZ010000129.1	ND
Escherichia coli strain 2018-226 STEC2018-226_contig00117, whole genome shotgun sequence	JAEAMV010000117.1	ND
Escherichia coli strain 1384-03 1384-03_contig00133, whole genome shotgun sequence	JAEAMT010000133.1	ND
Escherichia coli strain P17-291 P17-291_contig00069, whole genome shotgun sequence	JAEANK010000069.1	Eru7
Escherichia coli strain S18-168 S18-168_contig00068, whole genome shotgun sequence	JAEANJ010000068.1	Eru7
Escherichia coli strain S18-73 S18-73_contig00067, whole genome shotgun sequence	JAEANH010000067.1	Eru7
Escherichia coli strain S18-9-1 S18-9-1_contig00064, whole genome shotgun sequence	JAEANG010000064.1	Eru7
Escherichia coli strain S19-101-1 S19-101_contig00070, whole genome shotgun sequence	JAEANF010000070.1	Eru7
Escherichia coli strain S19-30-1 S19-30_contig00071, whole genome shotgun sequence	JAEANC010000071.1	Eru7
Escherichia coli strain S19-615-1 S19-615_contig00066, whole genome shotgun sequence	JAEANB010000066.1	Eru7
Escherichia coli strain S19-710-1 S19-710_contig00078, whole genome shotgun sequence	JAEAMY010000078.1	Eru7
Escherichia coli strain 2017-299-1 STEC2017-299-1_contig00071, whole genome shotgun sequence	JAEAMX010000071.1	Eru7
Escherichia coli strain 2017-353-1 STEC2017-353-1_contig00071, whole genome shotgun sequence	JAEAMW010000071.1	Eru7
Escherichia coli strain 2018-439 STEC2018-439_contig00071, whole genome shotgun sequence	JAEAMU010000071.1	Eru7
**PRJNA666781<br>STEC strains isolated from semi-hard raw milk cheese from Italy**		
**Strain**	**NCBI Accession no**	**Eru type**
**Stx2**		
Escherichia coli strain UC4128 STEC_UC4128_contig_75, whole genome shotgun sequence	JACZIB010000075.1	ND
Escherichia coli strain UC4130 STEC_UC4130_contig_74, whole genome shotgun sequence	JACZHZ010000074.1	ND
Escherichia coli strain UC4131 STEC_UC4131_contig_186, whole genome shotgun sequence	JACZHY010000186.1	ND
Escherichia coli strain UC4132 STEC_UC4132_contig_74, whole genome shotgun sequence	JACZHX010000074.1	ND
Escherichia coli strain UC4129 STEC_UC4129_contig_26, whole genome shotgun sequence	JACZIA010000026.1	ND
Escherichia coli strain UC4133 STEC_UC4133_contig_57, whole genome shotgun sequence	JACZHW010000057.1	ND
Escherichia coli strain UC4134 STEC_UC4134_contig_28, whole genome shotgun sequence	JACZHV010000028.1	ND
**Stx1**		
Escherichia coli strain UC4128 STEC_UC4128_contig_17, whole genome shotgun sequence	JACZIB010000017.1	Eru7
Escherichia coli strain UC4130 STEC_UC4130_contig_17, whole genome shotgun sequence	JACZHZ010000017.1	Eru7
Escherichia coli strain UC4131 STEC_UC4131_contig_23, whole genome shotgun sequence	JACZHY010000023.1	Eru7
Escherichia coli strain UC4132 STEC_UC4132_contig_17, whole genome shotgun sequence	JACZHX010000017.1	Eru7
**PRJNA248042<br>National Surveillance of STEC O157:H7 in England**		
**Strain**	**NCBI Accession no**	**Eru type**
**Stx2**		
Escherichia coli strain H121560360 SAMN03492009-rid9085603.guided.356, whole genome shotgun sequence	AAVVNG010000004.1	Eru1
Escherichia coli strain H121560360 SAMN03492009-rid9085603.denovo.333, whole genome shotgun sequence	AAVVVC010000003.1	Eru1

Escherichia coli strain H132920427 SAMN03485820-rid9085393.guided.134, whole genome shotgun sequence	AAVVMM010000010.1	Eru2
Escherichia coli strain H121780072 SAMN03492023-rid9085623.guided.351, whole genome shotgun sequence	AAVVLZ010000015.1	Eru7
Escherichia coli strain H053000190 SAMN03491912-rid9085593.guided.151, whole genome shotgun sequence	AAVVMI010000016.1	Eru2
Escherichia coli strain H054440305 SAMN03492026-rid9085653.guided.207, whole genome shotgun sequence	AAVVQR010000015.1	Eru2
Escherichia coli strain H063100370 SAMN03492028-rid9085673.guided.184, whole genome shotgun sequence	AAVVUX010000016.1	Eru7
Escherichia coli strain H063100370 SAMN03492028-rid9085673.denovo.134, whole genome shotgun sequence	AAVVMS010000018.1	Eru7
Escherichia coli strain H094000293 SAMN03492031-rid9085703.guided.352, whole genome shotgun sequence	AAVVOA010000015.1	Eru7
Escherichia coli strain H102620398 SAMN03492030-rid9085693.guided.275, whole genome shotgun sequence	AAVVVF010000015.1	Eru7
Escherichia coli strain H094000312 SAMN03492036-rid9085733.guided.276, whole genome shotgun sequence	AAVVXZ010000026.1	Eru7
Escherichia coli strain H094000312 SAMN03492036-rid9085733.denovo.205, whole genome shotgun sequence	AAVVNS010000026.1	Eru7
Escherichia coli strain H063160363 SAMN03492043-rid9085743.guided.172, whole genome shotgun sequence	AAVVJN010000025.1	Eru7
Escherichia coli strain H123780462 SAMN03492044-rid9085793.guided.166, whole genome shotgun sequence	AAVVRM010000028.1	Eru7
Escherichia coli strain H122440404 SAMN03492048-rid9085833.guided.183, whole genome shotgun sequence	AAVVKN010000025.1	Eru7
Escherichia coli strain H101980207 SAMN03492054-rid9085873.guided.281, whole genome shotgun sequence	AAVVKJ010000028.1	Eru7
Escherichia coli strain H043240557 SAMN03492051-rid9085843.guided.166, whole genome shotgun sequence	AAVVVB010000025.1	Eru7
Escherichia coli strain H123760762 SAMN03492056-rid9085893.guided.229, whole genome shotgun sequence	AAVVTZ010000010.1	Eru7
Escherichia coli strain H123900440 SAMN03492059-rid9085923.guided.177, whole genome shotgun sequence	AAVVXJ010000022.1	Eru7
Escherichia coli strain H052720465 SAMN03492060-rid9085933.guided.155, whole genome shotgun sequence	AAVVSU010000026.1	Eru7
Escherichia coli strain H091920551 SAMN03492063-rid9085963.guided.147, whole genome shotgun sequence	AAVVKR010000027.1	Eru7
Escherichia coli strain H123800424 SAMN03492066-rid9085993.guided.242, whole genome shotgun sequence	AATHXA010000027.1	Eru7
Escherichia coli strain H123180903 SAMN03492072-rid9086023.guided.415, whole genome shotgun sequence	AAVVGX010000027.1	Eru7
Escherichia coli strain H123820316 SAMN03492078-rid9086073.guided.372, whole genome shotgun sequence	AAVVVU010000025.1	Eru7
Escherichia coli strain H112340317 SAMN03492067-rid9086003.guided.165, whole genome shotgun sequence	AAVVSL010000029.1	Eru1
Escherichia coli strain H121620527 SAMN03492083-rid9086123.guided.165, whole genome shotgun sequence	AAVVMJ010000033.1	Eru1
Escherichia coli strain H122880422 SAMN03492081-rid9086103.guided.165, whole genome shotgun sequence	AAVVDW010000029.1	Eru1
Escherichia coli strain H122460748 SAMN03492087-rid9086143.guided.166, whole genome shotgun sequence	AAVVRE010000037.1	Eru1
Escherichia coli strain H120540363 SAMN03492095-rid9086193.guided.169, whole genome shotgun sequence	AAVVTU010000036.1	Eru1
Escherichia coli strain H123400303 SAMN03492107-rid9086273.guided.290, whole genome shotgun sequence	AAVVFF010000037.1	Eru1
Escherichia coli strain H122920156 SAMN03492121-rid9086373.guided.438, whole genome shotgun sequence	AATHXD010000066.1	Eru1
Escherichia coli strain H102820427 SAMN03492123-rid9086393.guided.210, whole genome shotgun sequence	AATHXU010000060.1	Eru1
Escherichia coli strain H121620525 SAMN03492115-rid9086343.guided.168, whole genome shotgun sequence	AAVVTX010000050.1	Eru5
Escherichia coli strain H093940423 SAMN03492129-rid9086443.guided.195, whole genome shotgun sequence	AAVVMY010000048.1	Eru7
Escherichia coli strain H132040221 SAMN03492131-rid9086463.guided.169, whole genome shotgun sequence	AAVVTC010000053.1	Eru1
Escherichia coli strain H123960525 SAMN03492134-rid9086493.guided.198, whole genome shotgun sequence	AAVVLU010000073.1	Eru1

Escherichia coli strain H121320380 SAMN03492135-rid9086503.guided.140, whole genome shotgun sequence	AAVVOD010000067.1	Eru1
Escherichia coli strain H121320380 SAMN03492135-rid9086503.denovo.082, whole genome shotgun sequence	AATHWY010000053.1	Eru7
Escherichia coli strain H122900266 SAMN03492148-rid9086603.guided.215, whole genome shotgun sequence	AAVVQZ010000056.1	Eru7
Escherichia coli strain H123480455 SAMN03492142-rid9086553.guided.197, whole genome shotgun sequence	AAVVED010000052.1	Eru5
Escherichia coli strain H123740302 SAMN03492155-rid9086673.guided.274, whole genome shotgun sequence	AAVVTC010000107.1	Eru1
Escherichia coli strain H123540543 SAMN03492158-rid9086703.guided.208, whole genome shotgun sequence	AAVVWC010000072.1	Eru7
Escherichia coli strain H121600272 SAMN03492156-rid9086683.guided.223, whole genome shotgun sequence	AAVVIM010000068.1	Eru7
Escherichia coli strain H123680777 SAMN03492169-rid9086783.guided.153, whole genome shotgun sequence	AAVVJS010000064.1	Eru1
Escherichia coli strain H121980151 SAMN03492170-rid9086793.guided.159, whole genome shotgun sequence	AAVVFK010000056.1	Eru1
Escherichia coli strain H122860601 SAMN03492171-rid9086803.guided.179, whole genome shotgun sequence	AAVVXH010000123.1	Eru7
Escherichia coli strain H122920157 SAMN03492172-rid9086813.guided.176, whole genome shotgun sequence	AAVVEV010000056.1	Eru1
Escherichia coli strain H134240606 SAMN03492175-rid9086843.guided.135, whole genome shotgun sequence	AAVVCY010000055.1	Eru1
Escherichia coli strain H134240606 SAMN03492175-rid9086843.denovo.113, whole genome shotgun sequence	AAVVFQ010000059.1	Eru1
Escherichia coli strain H122220862 SAMN03492174-rid9086833.guided.158, whole genome shotgun sequence	AAVVPS010000058.1	Eru1
Escherichia coli strain H131980146 SAMN03492180-rid9086873.guided.160, whole genome shotgun sequence	AAVVJC010000072.1	Eru1
Escherichia coli strain H122140365 SAMN03492185-rid9086923.guided.176, whole genome shotgun sequence	AAVVKG010000054.1	Eru2
Escherichia coli strain H121100178 SAMN03492188-rid9086953.guided.106, whole genome shotgun sequence	AAVVPA010000065.1	Eru1
Escherichia coli strain H121100178 SAMN03492188-rid9086953.denovo.051, whole genome shotgun sequence	AAVVHE010000064.1	Eru1
Escherichia coli strain H121620528 SAMN03492190-rid9086973.guided.173, whole genome shotgun sequence	AAVVHY010000075.1	ND
Escherichia coli strain H093700590 SAMN03492192-rid9086993.guided.171, whole genome shotgun sequence	AAVVGZ010000088.1	ND
Escherichia coli strain H122460420 SAMN03492195-rid9087023.guided.248, whole genome shotgun sequence	AAVVRJ010000077.1	Eru7
Escherichia coli strain H121820144 SAMN03492208-rid9087123.guided.155, whole genome shotgun sequence	AAVVWR010000124.1	Eru2
**Stx1**		
Escherichia coli strain H125180252 SAMN03492074-rid9086033.denovo.004, whole genome shotgun sequence	AAVVXL010000004.1	eru4
Escherichia coli strain E1653600 SAMN03703076-rid9072093.denovo.004, whole genome shotgun sequence	AATHWC010000004.1	Eru13
Escherichia coli strain H131620832 SAMN03492568-rid9038543.denovo.007, whole genome shotgun sequence	AAVVNQ010000007.1	lambdoid
Escherichia coli strain H132360377 SAMN03492323-rid9087503.denovo.001, whole genome shotgun sequence	AAVVSB010000001.1	lambdoid
Escherichia coli strain H133040516 SAMN03492437-rid9037593.denovo.004, whole genome shotgun sequence	AAVVPQ010000004.1	eru4
Escherichia coli strain H103460518 SAMN03492465-rid9037983.denovo.006, whole genome shotgun sequence	AAVVOT010000006.1	lambdoid
Escherichia coli strain H113320446 SAMN03492141-rid9086543.denovo.064, whole genome shotgun sequence	AAVVVG010000064.1	Eru1
Escherichia coli strain H130960185 SAMN03492409-rid9087913.denovo.075, whole genome shotgun sequence	AAVVQM010000074.1	Eru1
Escherichia coli strain E113096 SAMN03703088-rid9072583.denovo.051, whole genome shotgun sequence	AATHVY010000051.1	lambdoid
Escherichia coli strain E1688150 SAMN03702926-rid9033583.denovo.059, whole genome shotgun sequence	AATIBF010000058.1	lambdoid
Escherichia coli strain H103780083 SAMN03492786-rid9061363.denovo.051, whole genome shotgun sequence	AAVVJM010000050.1	Eru11

Escherichia coli strain H122220222 SAMN03492104-rid9086243.denovo.054, whole genome shotgun sequence	AAVVWI010000053.1	lambdoid
Escherichia coli strain H123000125 SAMN03492075-rid9086043.denovo.061, whole genome shotgun sequence	AAVVXF010000060.1	lambdoid
Escherichia coli strain H134660555 SAMN03702957-rid9033863.denovo.051, whole genome shotgun sequence	AATIAB010000050.1	lambdoid
Escherichia coli strain H122980845 SAMN03492153-rid9086653.denovo.106, whole genome shotgun sequence	AAVVUY010000106.1	lambdoid
Escherichia coli strain WX011665S01E SAMN03496101-rid9061543.denovo.055, whole genome shotgun sequence	AAVVGH010000054.1	lambdoid
Escherichia coli strain H123340502 SAMN03492674-rid9039133.denovo.051, whole genome shotgun sequence	AAVVMC010000050.1	lambdoid
Escherichia coli strain H122960360 SAMN03492359-rid9087693.denovo.092, whole genome shotgun sequence	AAVVRI010000092.1	lambdoid
Escherichia coli strain H123980865 SAMN03492202-rid9087063.denovo.054, whole genome shotgun sequence	AAVVTR010000053.1	lambdoid
Escherichia coli strain H122760538 SAMN03492653-rid9038943.denovo.066, whole genome shotgun sequence	AAVVMW010000065.1	lambdoid
Escherichia coli strain WX016375S01E SAMN03496188-rid9080733.denovo.051, whole genome shotgun sequence	AAVVDM010000050.1	lambdoid
Escherichia coli strain WX017993S01E SAMN03496184-rid9080543.denovo.052, whole genome shotgun sequence	AAVVEW010000051.1	lambdoid
Escherichia coli strain H123040570 SAMN03492632-rid9038833.denovo.093, whole genome shotgun sequence	AAVVHC010000093.1	lambdoid
Escherichia coli strain H123120512 SAMN03492068-rid9086013.denovo.074, whole genome shotgun sequence	AAVVXJ010000073.1	lambdoid
Escherichia coli strain H123200240 SAMN03492225-rid9087283.denovo.052, whole genome shotgun sequence	AAVVSS010000051.1	lambdoid
Escherichia coli strain H124440480 SAMN03492126-rid9086423.denovo.052, whole genome shotgun sequence	AAVVVT010000051.1	ND
Escherichia coli strain H104620471 SAMN03492154-rid9086663.denovo.062, whole genome shotgun sequence	AAVVUS010000061.1	lambdoid
Escherichia coli strain H102980650 SAMN03492463-rid9037963.denovo.060, whole genome shotgun sequence	AAVVOS010000060.1	lambdoid
Escherichia coli strain H132080461 SAMN03492034-rid9085713.denovo.065, whole genome shotgun sequence	AAVVYL010000064.1	lambdoid
Escherichia coli strain H122440402 SAMN03492475-rid9038083.denovo.055, whole genome shotgun sequence	AAVVOH010000054.1	lambdoid
Escherichia coli strain H121800664 SAMN03702960-rid9033893.denovo.060, whole genome shotgun sequence	AATIAA010000059.1	lambdoid
Escherichia coli strain H113580158 SAMN03492105-rid9086253.denovo.052, whole genome shotgun sequence	AAVVWF010000051.1	lambdoid
Escherichia coli strain H121380674 SAMN03492111-rid9086303.denovo.068, whole genome shotgun sequence	AAVVWD010000068.1	lambdoid
Escherichia coli strain WX016320S01E SAMN03496190-rid9080753.denovo.050, whole genome shotgun sequence	AAVVDJ010000049.1	lambdoid
Escherichia coli strain H123740300 SAMN03492626-rid9038773.denovo.058, whole genome shotgun sequence	AAVVHJ010000057.1	lambdoid
Escherichia coli strain E1753820 SAMN03703008-rid9034313.denovo.054, whole genome shotgun sequence	AATHYI010000053.1	lambdoid
Escherichia coli strain H121620536 SAMN03492418-rid9088013.denovo.089, whole genome shotgun sequence	AAVVQC010000089.1	lambdoid
Escherichia coli strain H130740146 SAMN03492441-rid9037623.denovo.054, whole genome shotgun sequence	AAVVPM010000053.1	lambdoid
Escherichia coli strain H132480192 SAMN03492045-rid9085803.denovo.070, whole genome shotgun sequence	AAVVYG010000069.1	lambdoid
Escherichia coli strain H123880634 SAMN03492052-rid9085853.denovo.096, whole genome shotgun sequence	AAVVYD010000096.1	lambdoid
Escherichia coli strain H122980190 SAMN03492442-rid9037633.denovo.124, whole genome shotgun sequence	AAVVPO010000124.1	lambdoid
Escherichia coli strain H123840423 SAMN03492519-rid9038683.denovo.069, whole genome shotgun sequence	AAVVHS010000069.1	lambdoid
Escherichia coli strain H121100178 SAMN03492188-rid9086953.denovo.054, whole genome shotgun sequence	AAVVTX010000053.1	lambdoid
Escherichia coli strain H132940671 SAMN03492649-rid9038913.denovo.083, whole genome shotgun sequence	AAVVNC010000081.1	Eru1
Escherichia coli strain H133040516 SAMN03492437-rid9037593.guided.073, whole genome shotgun sequence	AAVVPQ010000128.1	Eru4

Escherichia coli strain E1745770 SAMN03702990-rid9034143.denovo.061, whole genome shotgun sequence	AATHZE010000060.1	ND
Escherichia coli strain E1747730 SAMN03702922-rid9033553.denovo.063, whole genome shotgun sequence	AATIBI010000062.1	lambdoid
Escherichia coli strain H130300235 SAMN03492425-rid9033073.denovo.183, whole genome shotgun sequence	AAVVQB010000182.1	ND

PRJNA715185
Diversity of STEC in flour from Germany

Strain	NCBI Accession no	Eru type
**Stx2**		
Escherichia coli strain BfR-EC-17777 60, whole genome shotgun sequence	JAGEWD010000060.1	ND
Escherichia coli strain BfR-EC-17374 67, whole genome shotgun sequence	JAGEXU010000067.1	ND
Escherichia coli strain BfR-EC-17741 53, whole genome shotgun sequence	JAGEWM010000053.1	ND
Escherichia coli strain BfR-EC-17656 61, whole genome shotgun sequence	JAGEXI010000061.1	ND
Escherichia coli strain BfR-EC-17705 59, whole genome shotgun sequence	JAGEWZ010000059.1	ND
Escherichia coli strain BfR-EC-17730 55, whole genome shotgun sequence	JAGEWO010000055.1	ND
Escherichia coli strain BfR-EC-17709 60, whole genome shotgun sequence	JAGEWY010000060.1	ND
Escherichia coli strain BfR-EC-17710 59, whole genome shotgun sequence	JAGEWX010000059.1	ND
Escherichia coli strain BfR-EC-17722 59, whole genome shotgun sequence	JAGEWS010000059.1	ND
Escherichia coli strain BfR-EC-17655 58, whole genome shotgun sequence	JAGEXJ010000058.1	ND
Escherichia coli strain BfR-EC-17679 44, whole genome shotgun sequence	JAGEXB010000044.1	Eru9
Escherichia coli strain BfR-EC-17841 contig00037, whole genome shotgun sequence	JAGEVW010000037.1	Eru9
Escherichia coli strain BfR-EC-17718 47, whole genome shotgun sequence	JAGEWW010000047.1	ND
Escherichia coli strain BfR-EC-17719 47, whole genome shotgun sequence	JAGEWV010000047.1	ND
Escherichia coli strain BfR-EC-17680 51, whole genome shotgun sequence	JAGEXA010000051.1	Eru9
Escherichia coli strain BfR-EC-17678 39, whole genome shotgun sequence	JAGEXC010000039.1	Eru9
Escherichia coli strain BfR-EC-17778 35, whole genome shotgun sequence	JAGEWC010000035.1	ND
Escherichia coli strain BfR-EC-17779 34, whole genome shotgun sequence	JAGEWB010000034.1	ND
Escherichia coli strain BfR-EC-17387 9, whole genome shotgun sequence	JAGEXQ010000009.1	Eru9
**Stx1**		
Escherichia coli strain BfR-EC-17379 contig00014, whole genome shotgun sequence	JAGEXT010000014.1	ND
Escherichia coli strain BfR-EC-17386 contig00011, whole genome shotgun sequence	JAGEXR010000011.1	ND
Escherichia coli strain BfR-EC-17751 contig00012, whole genome shotgun sequence	JAGEWI010000012.1	ND
Escherichia coli strain BfR-EC-17856 contig00002, whole genome shotgun sequence	JAGEVS010000002.1	ND
Escherichia coli strain BfR-EC-17721 contig00001, whole genome shotgun sequence	JAGEWT010000001.1	ND
Escherichia coli strain BfR-EC-17380 contig00001, whole genome shotgun sequence	JAGEXS010000001.1	ND

PRJNA643688
Genome Sequences of twelve Shiga Toxin-Producing *Escherichia coli* Strains Isolated from dairy cattle in Portugal

Strain	NCBI Accession no	Eru type
**Stx2**		
Escherichia coli strain E7N3P7C8A Contig_26_consensus_sequence, whole genome shotgun sequence	JACBWM010000026.1	Eru1

Escherichia coli strain E7N8P4C1 Contig_42_consensus_sequence, whole genome shotgun sequence	JACBWI010000042.1	ND
Escherichia coli strain E7N15P4C10 Contig_2_consensus_sequence, whole genome shotgun sequence	JACBWG010000002.1	Eru7
Escherichia coli strain E7N18P5C8G Contig_17_consensus_sequence, whole genome shotgun sequence	JACBWE010000017.1	Eru8
Escherichia coli strain E7N6P4C8A Contig_40_consensus_sequence, whole genome shotgun sequence	JACBWL010000040.1	ND
Escherichia coli strain E7N6P4C8C Contig_53_consensus_sequence, whole genome shotgun sequence	JACBWK010000053.1	ND
Escherichia coli strain E7N6P4C8F Contig_38_consensus_sequence, whole genome shotgun sequence	JACBWJ010000038.1	ND
Escherichia coli strain E7N16P4C9 Contig_58_consensus_sequence, whole genome shotgun sequence	JACBWF010000058.1	ND
Escherichia coli strain E7N6P4C8A Contig_34_consensus_sequence, whole genome shotgun sequence	JACBWL010000034.1	ND
Escherichia coli strain E7N16P4C9 Contig_43_consensus_sequence, whole genome shotgun sequence	JACBWF010000043.1	ND
Escherichia coli strain E7N8P4C1 Contig_5_consensus_sequence, whole genome shotgun sequence	JACBWI010000005.1	Eru8
Escherichia coli strain E7N6P4C8C Contig_36_consensus_sequence, whole genome shotgun sequence	JACBWK010000036.1	ND
Escherichia coli strain E7N6P4C8C Contig_59_consensus_sequence, whole genome shotgun sequence	JACBWK010000059.1	ND
Escherichia coli strain E7N16P4C9 Contig_59_consensus_sequence, whole genome shotgun sequence	JACBWF010000059.1	ND
Escherichia coli strain E7N6P4C8F Contig_63_consensus_sequence, whole genome shotgun sequence	JACBWJ010000063.1	ND
**Stx1**		
Escherichia coli strain E7N18P5C8G Contig_11_consensus_sequence, whole genome shotgun sequence	JACBWE010000011.1	Eru7
Escherichia coli strain E7N6P4C8A Contig_12_consensus_sequence, whole genome shotgun sequence	JACBWL010000012.1	Eru6
Escherichia coli strain E7N6P4C8C Contig_24_consensus_sequence, whole genome shotgun sequence	JACBWK010000024.1	Eru6
Escherichia coli strain E7N6P4C8F Contig_36_consensus_sequence, whole genome shotgun sequence	JACBWJ010000036.1	ND
Escherichia coli strain E7N16P4C9 Contig_28_consensus_sequence, whole genome shotgun sequence	JACBWF010000028.1	Eru6
Escherichia coli strain E7V12P1C4G Contig_79_consensus_sequence, whole genome shotgun sequence	JACBWN010000079.1	ND
Escherichia coli strain E7N3P7C8A Contig_56_consensus_sequence, whole genome shotgun sequence	JACBWM010000056.1	lambdoid
Escherichia coli strain E7V3P1C1 Contig_7_consensus_sequence, whole genome shotgun sequence	JACBWP010000007.1	ND
Escherichia coli strain E7V4P1C10 Contig_30_consensus_sequence, whole genome shotgun sequence	JACBWO010000030.1	ND
Escherichia coli strain E7N12P2C4 Contig_71_consensus_sequence, whole genome shotgun sequence	JACBWH010000071.1	ND
**PRJNA438214**<br>**Draft genomes sequences of 18 *Escherichia coli* STEC strains from Switzerland**		
**Strain**	**NCBI Accession no**	**Eru type**
**Stx2**		
Escherichia coli strain 364060-17 NODE_89_length_17522_cov_9.483185, whole genome shotgun sequence	PYSE01000089.1	Eru7
Escherichia coli strain 364062-17 NODE_5_length_99620_cov_14.092529, whole genome shotgun sequence	PYSC01000005.1	Eru7
Escherichia coli strain 364064-17 NODE_95_length_16865_cov_12.828414, whole genome shotgun sequence	PYSA01000095.1	Eru7
Escherichia coli strain 364068-17 NODE_93_length_15220_cov_19.738422, whole genome shotgun sequence	PYRX01000093.1	Eru7
Escherichia coli strain 364073-17 NODE_64_length_24383_cov_39.192117, whole genome shotgun sequence	PYRU01000064.1	Eru7
Escherichia coli strain 364082-17 NODE_100_length_13439_cov_15.958609, whole genome shotgun sequence	PYRR01000100.1	Eru7
Escherichia coli strain 364072-17 scaffold_0186, whole genome shotgun sequence	PYRP01000186.1	ND
Escherichia coli strain 364079-17 scaffold_0171, whole genome shotgun sequence	PYRO01000171.1	ND

Escherichia coli strain 364059-17 NODE_126_length_11966_cov_12.106850, whole genome shotgun sequence	PYSF01000126.1	Eru7
Escherichia coli strain 364061-17 NODE_88_length_15513_cov_19.573573, whole genome shotgun sequence	PYSD01000088.1	Eru7
Escherichia coli strain 364063-17 scaffold_0125, whole genome shotgun sequence	PYSB01000125.1	Eru7
Escherichia coli strain 364067-17 scaffold_0011, whole genome shotgun sequence	PYRY01000011.1	Eru7
Escherichia coli strain 364069-17 NODE_71_length_15513_cov_65.140517, whole genome shotgun sequence	PYRW01000071.1	Eru7
Escherichia coli strain 364070-17 scaffold_0017, whole genome shotgun sequence	PYRV01000017.1	Eru7
Escherichia coli strain 364075-17 NODE_73_length_15513_cov_42.632068, whole genome shotgun sequence	PYRT01000073.1	Eru7
Escherichia coli strain 364077-17 NODE_88_length_15513_cov_17.408748, whole genome shotgun sequence	PYRS01000088.1	Eru7
Escherichia coli strain 364071-17 scaffold_0089, whole genome shotgun sequence	PYRQ01000089.1	Eru7
Escherichia coli strain 364066-17 scaffold_0737, whole genome shotgun sequence	PYRZ01000737.1	ND
Escherichia coli strain 364066-17 scaffold_0504, whole genome shotgun sequence	PYRZ01000504.1	ND
