# Supplementary material for: High diversity in the regulatory region of Shiga toxin encoding bacteriophages
Source: BMC Genomics. 2022 Mar 24;23:230. doi: 10.1186/s12864-022-08428-5 (PMC8951638; doi:10.1186/s12864-022-08428-5)
Supplement: Supplementary file 3 — Additional file 3. Information about the 260 CI sequences used in phylogenetic analysis [file 12864_2022_8428_MOESM3_ESM.pdf]

Additional file 3

Information about the 260 CI sequences used in phylogenetic analysis

CI ID (NCBI)	Phage/Strain	Serotype	NCBI ACCESSION NO	Origin	Year	Stx type	Eru type
ACI32363.1	Enterobacteria phage YYZ-2008	O157:H7	FJ184280	Canada	2008	Stx1	Eru2
ACI43117.1	Stx2 Converting phage 1717	O157:H7	FJ188381	Canada	2008	Stx2	Eru2
ADN68413.1	Stx2 converting phage vB_EcoP_24B	O157:H7	HM208303.1	UK	2010	Stx2	Eru5
AHZ95179.1	Shigella phage POCJ13		KJ603229.1	USA	2014	Stx1	Eru4
AIF74338.1	Escherichia Stx1-converting recombinant phage HUN/2013	O157:H7	KJ909655.1	Hungary	2013	Stx1	Eru3
AKI86003.1	Escherichia phage PA4	O157:H7	KP682372.1	USA	2015	Stx2	Eru3
AKI86096.1	Escherichia phage PA5	O157:H7	KP682373.1	USA	2015	Stx2	Eru3
AKI86274.1	Escherichia phage PA11	O157:H7	KP682375.1	USA	2015	Stx2	Eru3
AKI86367.1	Escherichia phage PA16	O157:H7	KP682377.1	USA	2015	Stx2	Eru3
AKI86553.1	Escherichia phage PA21	O157:H7	KP682379.1	USA	2015	Stx2	Eru3
AKI86648.1	Escherichia phage PA27	O157:H7	KP682380.1	USA	2015	Stx2	Eru3
AKI86748.1	Escherichia phage PA28	O157:H7	KP682381.1	USA	2015	Stx2	lambdoid
AKI86834.1	Escherichia phage PA29	O157:H7	KP682382.1	USA	2015	Stx2	Eru3
AKI87206.1	Escherichia phage PA36	O157:H7	KP682386.1	USA	2015	Stx2	Eru3
AKI87302.1	Escherichia phage PA42	O157:H7	KP682387.1	USA	2015	Stx2	Eru3
AKI87492.1	Escherichia phage PA45	O157:H7	KP682389.1	USA	2015	Stx2	Eru3
AKI87588.1	Escherichia phage PA50	O157:H7	KP682390.1	USA	2015	Stx2	Eru3
AKI87680.1	Escherichia phage PA52	O157:H7	KP682392.1	USA	2015	Stx2	Eru3
AKJ74710.1	Escherichia phage PA12	O157:H7	KP682376.1	USA	2015	Stx2	Eru3
ANJ63820.1	Stx1 converting phage AU5Stx1	O157	KU977419.1	Australia	2016	Stx1	lambdoid
ANJ63898.1	Stx1 converting phage AU6Stx1	O157	KU977420.1	Australia	2016	Stx1	lambdoid
AVD99089.1	Escherichia phage GER2	O117:H7	MG710528.1	UK	2017	Stx1	Eru1
BAB87968.1	Stx2 Converting phage I	O157:H7	AP004402	Japan	2001	Stx2	lambdoid
BAC77937.1	Morioka V526	O157:H7	AP005153.1	Japan	2003	Stx1	Eru3
BAC78103.1	Morioka V526	O157:H7	AP005154.1	Japan		Stx2	Eru3
BAT31827.1	Stx2-converting phage Stx2a_F403 proviral	O157:H7	AP012529.1	Japan	2012	Stx2	Eru5
BAT31911.1	Stx2-converting phage Stx2a_F349	O157:H7	AP012530.1	Japan	2015	Stx2	Eru2
BAT32000.1	Stx2-converting phage Stx2a_F422 proviral	O157:H7	AP012531.1	Japan	2012	Stx2	lambdoid
BAT32093.1	Stx2-converting phage Stx2a_F451	O157:H8	AP012532.1	Japan	2012	Stx2	Eru5
BAT32177.1	Stx2-converting phage Stx2a_F723	O157:H9	AP012533.1	Japan	2012	Stx2	lambdoid
BAT32263.1	Stx2-converting phage Stx2a_F765 proviral	O157:H7	AP012534.1	Japan	2012	Stx2	Eru1
BAT32321.1	Stx2-converting phage Stx2a_WGPS9 proviral	O157:H7	AP012535.1	Japan	2012	Stx2	lambdoid
BAT32387.1	Stx2-converting phage Stx2a_1447 proviral	O157:H7	AP012536.1	Japan	2012	Stx2	Eru6
BAT32452.1	Stx2-converting phage Stx2a_WGPS2 proviral	O157:H7	AP012537.1	Japan	2012	Stx2	Eru6
BAT32531.1	Stx2-converting phage Stx2a_WGPS4 proviral	O157:H7	AP012538.1	Japan	2012	Stx2	Eru2
BAT32614.1	Stx2-converting phage Stx2a_WGPS6 proviral	O157:H7	AP012539.1	Japan	2012	Stx2	Eru2

BAT32693.1	Stx2-converting phage Stx2a_WGPS8 proviral	O157:H7	AP012540.1	Japan	2012	Stx2	Eru2
BCI48989.1	Stx2a-converting phage Stx2_14040	O145:H28	LC567818.1	Japan	2020	Stx2	Eru7
BCI49050.1	Stx1a-converting phage Stx1_14040	O145:H28	LC567819.1	Japan	2020	Stx1	Eru7
BCI49153.1	Stx2a-converting phage Stx2_14744	O145:H28	LC567820.1	Japan	2020	Stx2	Eru7
BCI49214.1	Stx1a-converting phage Stx1_14744	O145:H28	LC567821.1	Japan	2020	Stx1	Eru7
CAC83529.1	Enterobacteria phage phiP27	ONT:H-	AJ298298	Germany	2002	Stx2	Eru7
CAD88825.1	Phage BP-4795 complete genome	O84:H4	AJ556162.1	Germany	2003	Stx1	lambdoid
CAQ82008.1	Enterobacteria phage 2851	O157:H7	FM180578.1	Germany	1993	Stx2	Eru2
CCG06176.1	Escherichia phage P13374 proviral	O104:H4	HE664024.1	Germany	2011	Stx2	Eru1
CDK12686.1	Escherichia phage P13803	O2:H27	HG792102.1	Germany	2013	Stx2	Eru1
CDK23993.1	Escherichia phage P14437	O104:H4	HG792105.1	Norway	2006	Stx2	Eru1
CDL18842.1	Escherichia phage P13771	O104:H4	HG792104.1	Germany	2009	Stx2	Eru1
EEC26960.1	TW14588	O157:H7	ABKY02000003	USA	2006	Stx2	Eru3
EEC27821.1	TW14588	O157:H7	ABKY02000002	USA	2006	Stx2	Eru1
EFL4537683.1	E1653600	O157:H7	AATHWC010000004.1	UK		Stx1	Eru13
EFL4561882.1	E113096	O157:H7	AATHVY010000051.1	UK		Stx1	lambdoid
EFL4759493.1	H123800424	O157:H7	AATHXA010000027.1	UK		Stx2	Eru7
EFL4780259.1	H122920156	O157:H7	AATHXD010000066.1	UK		Stx2	Eru1
EFL4785786.1	H121320380	O157:H7	AATHWY010000053.1	UK		Stx2	Eru7
EFL4810920.1	H102820427	O157:H7	AATHXU010000060.1	UK		Stx2	Eru1
EFL4852155.1	E1753820	O157:H7	AATHYI010000053.1	UK		Stx1	lambdoid
EFL5060758.1	H121800664	O157:H7	AATIAA010000059.1	UK		Stx1	lambdoid
EFL5163126.1	H134660555	O157:H7	AATIAB010000050.1	UK		Stx1	lambdoid
EFL5223636.1	E1688150	O157:H7	AATIBF010000058.1	UK		Stx1	lambdoid
EGE5688604.1	H134240606	O157:H7	AAVVCY010000055.1	UK		Stx2	Eru2
EGE5822072.1	WX016320S01E	O157:H7	AAVVDJ010000049.1	UK		Stx1	lambdoid
EGE5827186.1	WX016375S01E	O157:H7	AAVVDM010000050.1	UK		Stx1	lambdoid
EGE5841739.1	H122880422	O157:H7	AAVVDW010000029.1	UK		Stx2	Eru1
EGE5909574.1	H123480455	O157:H7	AAVVED010000052.1	UK		Stx2	Eru5
EGE5954436.1	H122920157	O157:H7	AAVVEV010000056.1	UK		Stx2	Eru1
EGE5963960.1	H123400303	O157:H7	AAVVFF010000037.1	UK		Stx2	Eru1
EGE6051643.1	WX017993S01E	O157:H7	AAVVEW010000051.1	UK		Stx1	lambdoid
EGE6056760.1	H134240606	O157:H7	AAVVFQ010000059.1	UK		Stx2	Eru1
EGE6062049.1	H121980151	O157:H7	AAVVFK010000056.1	UK		Stx2	Eru1
EGE6148684.1	WX011665S01E	O157:H7	AAVVGH010000054.1	UK		Stx1	lambdoid
EGE6228835.1	H123180903	O157:H7	AAVVGX010000027.1	UK		Stx2	Eru7
EGE6276458.1	H121100178	O157:H7	AAVVHE010000064.1	UK		Stx2	Eru1
EGE6286513.1	H123040570	O157:H7	AAVVHC010000093.1	UK		Stx1	lambdoid
EGE6307131.1	H123740300	O157:H7	AAVVHJ010000057.1	UK		Stx1	lambdoid
EGE6419498.1	H123840423	O157:H7	AAVVHS010000069.1	UK		Stx1	lambdoid
EGE6460037.1	H121600272	O157:H7	AAVVIM010000068.1	UK		Stx2	Eru7

EGE6542355.1	H131980146	O157:H7	AAVVJC010000072.1	UK		Stx2	Eru1
EGE6605736.1	H063160363	O157:H7	AAVVJN010000025.1	UK		Stx2	Eru7
EGE6633054.1	H123680777	O157:H7	AAVVJS010000064.1	UK		Stx2	Eru1
EGE6689883.1	H122140365	O157:H7	AAVVKG010000054.1	UK		Stx2	Eru2
EGE6708400.1	H101980207	O157:H7	AAVVKJ010000028.1	UK		Stx2	Ery7
EGE6722855.1	H122440404	O157:H7	AAVVKN010000025.1	UK		Stx2	Eru7
EGE6749235.1	H091920551	O157:H7	AAVVKR010000027.1	UK		Stx2	Eru7
EGE6922782.1	H121780072	O157:H7	AAVVLZ010000015.1	UK		Stx2	Eru7
EGE6963583.1	H121620527	O157:H7	AAVVMJ010000033.1	UK		Stx2	Eru1
EGE6977926.1	H053000190	O157:H7	AAVVMI010000016.1	UK		Stx2	Eru2
EGE6982261.1	H132920427	O157:H7	AAVVMM010000010.1	UK		Stx2	Eru2
EGE7018974.1	H063100370	O157:H7	AAVVMS010000018.1	UK		Stx2	Eru7
EGE7050911.1	H093940423	O157:H7	AAVVMY010000048.1	UK		Stx2	Eru7
EGE7055905.1	H122760538	O157:H7	AAVVMW010000065.1	UK		Stx1	lambdoid
EGE7073250.1	H121560360	O157:H7	AAVVNG010000004.1	UK		Stx2	Eru1
EGE7134837.1	H131620832	O157:H7	AAVVNQ010000007.1	UK		Stx1	lambdoid
EGE7146195.1	H094000312	O157:H7	AAVVNS010000026.1	UK		Stx2	Eru7
EGE7166646.1	H094000293	O157:H7	AAVVOA010000015.1	UK		Stx2	Eru7
EGE7199773.1	H121320380	O157:H7	AAVVOD010000067.1	UK		Stx2	Eru1
EGE7230283.1	H122440402	O157:H7	AAVVOH010000054.1	UK		Stx1	lambdoid
EGE7333290.1	H121100178	O157:H7	AAVVPA010000065.1	UK		Stx2	Eru1
EGE7343382.1	H130740146	O157:H7	AAVVPM010000053.1	UK		Stx1	lambdoid
EGE7370760.1	H133040516	O157:H7	AAVVPQ010000004.1	UK		Stx1	Eru4
EGE7389752.1	H122220862	O157:H7	AAVVPS010000058.1	UK		Stx2	Eru1
EGE7394257.1	H122980190	O157:H7	AAVVPO010000124.1	UK		Stx1	lambdoid
EGE7456400.1	H121620536	O157:H7	AAVVQC010000089.1	UK		Stx1	lambdoid
EGE7512252.1	H130960185	O157:H7	AAVVQM010000074.1	UK		Stx1	Eru1
EGE7526414.1	H123880824	O157:H7	AAVVQR010000015.1	UK		Stx2	Eru2
EGE7568803.1	H122900266	O157:H7	AAVVQZ010000056.1	UK		Stx2	Eru7
EGE7603615.1	H122460748	O157:H7	AAVVRE010000037.1	UK		Stx2	Eru1
EGE7625108.1	H122960360	O157:H7	AAVVRI010000092.1	UK		Stx1	lambdoid
EGE7644261.1	H123780462	O157:H7	AAVVRM010000028.1	UK		Stx2	Eru7
EGE7696677.1	H132360377	O157:H7	AAVVSB010000001.1	UK		Stx1	lambdoid
EGE7750849.1	H112340317	O157:H7	AAVVSL010000029.1	UK		Stx2	Eru1
EGE7793292.1	H123200240	O157:H7	AAVVSS010000051.1	UK		Stx1	lambdoid
EGE7868356.1	H052720465	O157:H7	AAVVSU010000026.1	UK		Stx2	Eru7
EGE7880002.1	H124600634	O157:H7	AAVVTC010000107.1	UK		Stx2	Eru1
EGE7946210.1	H123980865	O157:H7	AAVVTR010000053.1	UK		Stx1	lambdoid
EGE7955773.1	H120540363	O157:H7	AAVVTU010000036.1	UK		Stx2	Eru1
EGE7978731.1	H123760762	O157:H7	AAVVTZ010000010.1	UK		Stx2	Eru7
EGE8029056.1	H121620525	O157:H7	AAVVTX010000050.1	UK		Stx2	Eru5

EGE8029099.1	H121100178	O157:H7	AAVVTX010000053.1	UK		Stx1	lambdoid
EGE8059842.1	H104620471	O157:H7	AAVVUS010000061.1	UK		Stx1	lambdoid
EGE8089196.1	H063100370	O157:H7	AAVVUX010000016.1	UK		Stx2	Eru7
EGE8094777.1	H122980845	O157:H7	AAVVUY010000106.1	UK		Stx1	lambdoid
EGE8105221.1	H113320446	O157:H7	AAVVVG010000064.1	UK		Stx1	Eru1
EGE8114167.1	H135020660	O157:H7	AAVVVF010000015.1	UK		Stx2	Eru7
EGE8120612.1	H043240557	O157:H7	AAVVVB010000025.1	UK		Stx2	Eru7
EGE8122814.1	H121560360	O157:H7	AAVVVC010000003.1	UK		Stx2	Eru1
EGE8201356.1	H123820316	O157:H7	AAVVVU010000025.1	UK		Stx2	Eru7
EGE8248496.1	H121380674	O157:H7	AAVVWD010000068.1	UK		Stx1	lambdoid
EGE8253777.1	H133400671	O157:H7	AAVVWC010000072.1	UK		Stx2	Eru7
EGE8259112.1	H113580158	O157:H7	AAVVWF010000051.1	UK		Stx1	lambdoid
EGE8285006.1	H122220222	O157:H7	AAVVWI010000053.1	UK		Stx1	lambdoid
EGE8319594.1	H132580296	O157:H7	AAVVWR010000124.1	UK		Stx2	Eru2
EGE8396302.1	H123000125	O157:H7	AAVVXF010000060.1	UK		Stx1	lambdoid
EGE8406126.1	H103680174	O157:H7	AAVVXH010000123.1	UK		Stx2	Eru7
EGE8409932.1	H123900440	O157:H7	AAVVXJ010000022.1	UK		Stx2	Eru7
EGE8411765.1	H123120512	O157:H7	AAVVXJ010000073.1	UK		Stx1	lambdoid
EGE8423942.1	H125180252	O157:H7	AAVVXL010000004.1	UK		Stx1	Eru4
EGE8497699.1	H094000312	O157:H7	AAVVXZ010000026.1	UK		Stx2	Eru7
EGE8529150.1	H123880634	O157:H7	AAVVYD010000096.1	UK		Stx1	lambdoid
EGE8534332.1	H132480192	O157:H7	AAVVYG010000069.1	UK		Stx1	lambdoid
EGE8565673.1	H132080461	O157:H7	AAVVYL010000064.1	UK		Stx1	lambdoid
EGE8879459.1	H103460518	O157:H7	AAVVOT010000006.1	UK		Stx1	lambdoid
EGE8936903.1	H102980650	O157:H7	AAVVOS010000060.1	UK		Stx1	lambdoid
KYR25636.1	STEC 731	O145:Hnt	LOFN01000117.1	Netherlands	2013	Stx2	Eru7
KYR31563.1	STEC 709	O26:H11	LOFM01000026.1	Netherlands	2013	Stx2	Eru7
KYR32735.1	STEC 709	O26:H11	LOFM01000001.1	Netherlands	2013	Stx2	Eru1
KYR47546.1	STEC 931	O26:H11	LOFS01000183.1	Netherlands	2013	Stx2	Eru1
KYR50760.1	STEC 886	O91:H14	LOFR01000041.1	Netherlands	2013	Stx1	Eru4
KYR81241.1	STEC 1188	O91:H14	LOFX01000035.1	Netherlands	2013	Stx1	Eru4
KYS15770.1	STEC 1293	O26:H11	LOGF01000197.1	Netherlands	2013	Stx2	Eru7
KYS20709.1	STEC 1299	O128:H2	LOGG01000109.1	Netherlands	2013	Stx1	Eru4
KYS26052.1	STEC1299	O128:H2	LOGG01000057.1	Netherlands	2013	Stx2	Eru6
KYS37089.1	STEC1375	O185:H7	LOGJ01000018.1	Netherlands	2013	Stx2	Eru7
KYS42165.1	STEC 1363	O128:H2	LOGI01000090.1	Netherlands	2013	Stx2	Eru6
KYS44558.1	STEC 1442	O145:Hnt	LOGK01000083.1	Netherlands	2014	Stx2	Eru7
KYS51717.1	STEC 1465	O128:H2	LOGL01000097.1	Netherlands	2014	Stx1	Eru1
KYS62574.1	STEC 1500	O76:H19	LOGN01000031.1	Netherlands	2014	Stx1	Eru4
KYS86001.1	STEC1686	O27:H30	LOGT01000177.1	Netherlands	2014	Stx2	Eru10
KYS86158.1	STEC 1585	O91:H14	LOGR01000002.1	Netherlands	2014	Stx1	Eru4

KYT16274.1	STEC2193	O103:H2	LOGW01000035.1	Netherlands	2013	Stx1	lambdoid
KYT16329.1	STEC 2236	O113:H4	LOGY01000073.1	Netherlands	2013	Stx2	lambdoid
KYT35599.1	STEC 1506	O5:H19	LPWV01000121.1	Netherlands	2014	Stx1	Eru4
KYT56146.1	STEC 2363	O91:H14	LPWY01000046.1	Netherlands	2013	Stx1	Eru4
KYT60009.1	STEC 2419	O103:H2	LPWZ01000120.1	Netherlands	2013	Stx1	lambdoid
KYT71597.1	STEC 2505	O128:H2	LPXB01000100.1	Netherlands	2013	Stx2	Eru6
KYT71713.1	STEC 2505	O128:H2	LPXB01000098.1	Netherlands	2013	Stx1	Eru4
KYT73689.1	STEC 2450	O146:H21	LPXA01000101.1	Netherlands	2013	Stx1	Eru4
KYT80232.1	STEC 2954	O174:H8	LPXE01000152.1	Netherlands	2013	Stx1	Eru4
KYT81410.1	STEC 2746	O128ab:H2	LPXD01000148.1	Netherlands	2013	Stx2	Eru6
KYT86498.1	STEC 2746	O128ab:H2	LPXD01000109.1	Netherlands	2013	Stx1	Eru4
KYT97710.1	STEC 2499	O181:H49	LOIE01000066.1	Netherlands	2013	Stx1	Eru7
KYU10111.1	STEC 2564	O117:H7	LOIG01000176.1	Netherlands	2013	Stx1	Eru1
KYU17220.1	STEC 2591	O174:H2	LOII01000032.1	Netherlands	2013	Stx1	Eru7
KYU24994.1	STEC 2633	O146:H10	LOIK01000066.1	Netherlands	2013	Stx1	Eru5
KYU38540.1	STEC 2764	O174:H8	LOIN01000127.1	Netherlands	2013	Stx1	Eru4
KYU47608.1	STEC 2788	O91:H14	LOIO01000033.1	Netherlands	2013	Stx1	Eru4
KYU60947.1	STEC 2841	O nt:H20	LOIQ01000003.1	Netherlands	2013	Stx1	Eru4
KYU68016.1	STEC 2894.1	O1:H20	LOIS01000032.1	Netherlands	2013	Stx1	Eru5
KYU87735.1	STEC2953	O113:H4	LOIW01000084.1	Netherlands	2016	Stx2	Eru10
KYU88777.1	STEC2953	O113:H4	LOIW01000078.1	Netherlands	2013	Stx1	Eru4
KYU89986.1	STEC 2980	O128ab:H2	LOIY01000194.1	Netherlands	2014	Stx1	Eru4
KYU92507.1	STEC 2962	O91:H14	LOIX01000034.1	Netherlands	2013	Stx1	Eru4
KYV00368.1	STEC2980	O128ab:H2	LOIY01000123.1	Netherlands	2014	Stx2	Eru6
KYV06485.1	STEC 3031	O38:H26	LOIZ01000129.1	Netherlands	2014	Stx2	Eru6
KYV07180.1	STEC 3031	O38:H26	LOIZ01000128.1	Netherlands	2014	Stx1	Eru4
KYV16898.1	STEC 2839	O76:H19	LOJB01000015.1	Netherlands	2013	Stx1	Eru4
KYV23489.1	STEC 2064	O91:H14	LOJC01000003.1	Netherlands	2013	Stx1	Eru4
KYV25047.1	STEC 2074	O146:H21	LOJD01000082.1	Netherlands	2013	Stx1	Eru4
KYV25655.1	STEC 66	O165:H25	LNFT01000172.1	Netherlands	2013	Stx2	Eru1
KYV36935.1	STEC 29	O91:H14	LNFU01000056.1	Netherlands	2013	Stx1	Eru4
KYV47051.1	STEC 196	O91:H14	LNZK01000067.1	Netherlands	2013	Stx1	Eru4
KYV51123.1	STEC 168	O91:H14	LNFV01000026.1	Netherlands	2013	Stx1	Eru4
KYV59879.1	STEC 200	O174:H21	LNZL01000033.1	Netherlands	2013	Stx2	Eru7
KYV68949.1	STEC 329	O91:H14	LOCT01000103.1	Netherlands	2013	Stx1	Eru4
KYW17099.1	STEC 559	O182:H2	LODC01000145.1	Netherlands	2013	Stx1	lambdoid
KYW31865.1	STEC 565	O121:H19	LODE01000106.1	Netherlands	2013	Stx2	Eru2
KYW38149.1	STEC 645	O91:H14	LODG01000045.1	Netherlands	2013	Stx1	Eru4
KYW59579.1	STEC 3084	O91:H14	LPUJ01000040.1	Netherlands		Stx1	Eru4
KYW63559.1	STEC 3087	O91:H14	LPUK01000020.1	Netherlands	2014	Stx1	Eru4
KYW70309.1	STEC 3106	O91:H14	LPUN01000125.1	Netherlands		Stx1	Eru4

KYW76135.1	STEC 3098	O174:H21	LPUM01000015.1	Netherlands	2014	Stx2	Eru6
MBH5127850.1	2018-439		JAEAMU010000071.1	Switzerland	2018	Stx2	Eru7
MBH5152383.1	2017-353-1		JAEAMW010000071.1	Switzerland	2017	Stx2	Eru7
MBH5157946.1	S19-710-1		JAEAMY010000078.1	Switzerland	2019	Stx2	Eru7
MBH5162990.1	2017-299-1		JAEAMX010000071.1	Switzerland	2017	Stx2	Eru7
MBH5178762.1	S19-30-1		JAEANC010000071.1	Switzerland	2019	Stx2	Eru7
MBH5194740.1	S19-18-1		JAEANE010000081.1	Switzerland	2019	Stx2	Eru7
MBH5199905.1	S19-101-1		JAEANF010000070.1	Switzerland	2019	Stx2	Eru7
MBH5205094.1	S18-73		JAEANH010000067.1	Switzerland	2018	Stx2	Eru7
MBH5215298.1	S18-9-1		JAEANG010000064.1	Switzerland	2018	Stx2	Eru7
MBH5225647.1	P17-291		JAEANK010000069.1	Switzerland	2017	Stx2	Eru7
MBH5239801.1	S18-168		JAEANJ010000068.1	Switzerland	2018	Stx2	Eru7
MBI1440405.1	STEC_UC4132		JACZHX010000017.1	Italy	2019	Stx1	Eru7
MBI1456586.1	STEC_UC4130		JACZHZ010000017.1	Italy		Stx1	Eru7
MBI1465333.1	STEC_UC4131		JACZHY010000023.1	Italy		Stx1	Eru7
MBI1490075.1	STEC_UC4128		JACZIB010000017.1	Italy		Stx1	Eru7
MBL6171648.1	LSC6-3		JAETZC010000029.1	Switzerland	2020	Stx2	Eru10
MBL6193510.1	LSC1-7		JAETZA010000009.1	Switzerland	2020	Stx1	Eru4
MBL6204510.1	ATC7-7		JAETYU010000018.1	Switzerland		Stx1	Eru4
MBL6236395.1	LSC1-58		JAETYZ010000032.1	Switzerland	2020	Stx1	Eru4
MBL6291528.1	LSC-5-20		JAETYY010000002.1	Switzerland	2020	Stx1	Eru4
MBL6338105.1	ATC36-6		JAETYQ010000045.1	Switzerland	2020	Stx1	Eru4
MBL6358120.1	ATC-4-67		JAETYK010000017.1	Switzerland	2020	Stx1	Eru1
MBL6374145.1	ATC-15-17		JAETYI010000026.1	Switzerland	2020	Stx1	Eru1
MBL6378896.1	ATB47-1		JAETYG010000030.1	Switzerland	2020	Stx2	lambdoid
MBL6401972.1	ATB-14-66		JAETYA010000007.1	Switzerland	2020	Stx1	Eru4
MBL6419043.1	ATB-10-31		JAETXY010000022.1	Switzerland	2020	Stx1	Eru4
MBL9224177.1	STEC 559	O182:H2	LODC01000145.1	Netherlands	2013	Stx1	lambdoid
NP_309212.2	Sakai	O157:H7	NC_002695	Japan	2000	Stx2	Eru3
NP_311017.2	Escherichia coli str. Sakai	O157:H7	NC_002695	Japan	2000	Stx1	lambdoid
NYR48883.1	E7N18P5C8G	ONT:H28	JACBWE010000011.1	Portugal	2019	Stx1	Eru7
NYR49962.1	E7N18P5C8G	ONT:H28	JACBWE010000017.1	Portugal	2019	Stx2	Eru8
NYR61457.1	E7N15P4C10	O116:H21	JACBWG010000002.1	Portugal	2019	Stx2	Eru7
NYR80058.1	E7N6P4C8C	O29:H12	JACBWK010000024.1	Portugal	2019	Stx1	Eru6
NYR83788.1	E7N6P4C8A	O29:H12	JACBWL010000012.1	Portugal	2019	Stx1	Eru6
NYS00014.1	E7N3P7C8A	O150:H2	JACBWM010000026.1	Portugal	2019	Stx2	Eru1
NYS00969.1	E7N3P7C8A	O150:H2	JACBWM010000056.1	Portugal	2019	Stx1	lambdoid
PAT83787.1	536-9	O26:H11	MRVS01000020.1	Israel	2016	Stx2	Eru7
PAT90662.1	479BS2	O26:H11	MRVR01000011.1	Israel	2016	Stx2	Eru7
PAT95399.1	476-14	O26:H11	MRVU01000049.1	Israel	2016	Stx2	Eru7
PAT99334.1	510-5	O26:H11	MRVV01000040.1	Israel	2016	Stx2	Eru7

PAU07863.1	625C-4	O26:H11	MRVW01000016.1	Israel	2016	Stx2	Eru7
PAU13589.1	514-2	O174:H21	MRVZ01000122.1	Israel	2016	Stx2	Eru7
PAU30050.1	573-4	O171:H29	MRWA01000015.1	Israel	2016	Stx2	Eru7
PRT57725.1	St. Olav164		PVRW01000040.1	Norway		Stx2	Eru7
QIW91713.1	Lys8385Vzw	O103:H11	MT225100	Japan	2020	Stx1	Eru6
QIW91773.1	Lys19259Vzw	O157:H7	MT225101	Japan	2020	Stx2	lambdoid
QKA14637.1	NE 1092-2	O157:H7	NZ_CP038328.1	USA	2000	Stx1	Eru1
QKA15101.1	NE 1092-2	O157:H7	CP038328.1	USA	2000	Stx2	Eru2
YP_001648917.1	Enterobacteria phage Min27	O157:H7	NC_010237.1	China	2007	Stx2	Eru5
YP_002274230.1	Stx2-converting phage 1717, complete prophage genome	O157:H7	NC_011357.1	Canada	2008	Stx2	Eru2
YP_009226843.1	Shigella phage 75/02 Stx	Shigella	NC_029120.1	Hungary	2013	Stx1	Eru4
YP_009907967.1	Escherichia phage SH2026Stx1	O157:H7	NC_049919.1	USA	2018	Stx1	Eru2
YP_007001447.1	TL-2011c	O103:H4	NC_019442	Norway	2006	Stx2	Eru1
AAD25430.1	933W	O157:H7	NC_000924	USA	1982	Stx2	lambdoid
NP_040628.1	lambda		NC_001416				lambdoid
JAGEXB010000044	BfR-EC-17679	O36:H14	JAGEXB010000044.1	Switzerland	2018	Stx2	Eru9
KYU24344.1	STEC 2595		LOIJ01000033.1	Netherlands	2013	Stx2	Eru11
CCVP01000073	FHI32		CCVP01000073.1	Norway	2009	Stx1	Eru12
YP_794110.1	Stx2-converting phage 86	O86:H-	NC_008464.1	Japan		Stx2	Eru3
