# Supplementary material for: High diversity in the regulatory region of Shiga toxin encoding bacteriophages
Source: BMC Genomics. 2022 Mar 24;23:230. doi: 10.1186/s12864-022-08428-5 (PMC8951638; doi:10.1186/s12864-022-08428-5)
Supplement: Supplementary file 4 — Additional file 4. Percent Identity Matrix (Clustal2.1) between clades of CI repressor sequences [file 12864_2022_8428_MOESM4_ESM.pdf]

Additional file 4

Percent Identity Matrix (Clustal2.1) between clades of CI repressor sequences.

	I	II	III	IV	V	VIa	VIb	VIIa	VIIIa
I	100	**51**	<25	<25	<25	<25	<25	<25	<25
II		100	<25	<25	<25	<25	<25	<25	<25
III			100	**60**	**32**	<25	<25	<25	<25
IV				100	**42**	<25	**35**	<25	<25
V					100	<25	<25	<25	<25
VIa						100	**37**	<25	<25
VIb							100	<25	<25
VII								100	<25
VIII									100
